# Development of a digital intervention for psychedelic preparation (DIPP)

**DOI:** 10.1038/s41598-024-54642-4

**Published:** 2024-02-19

**Authors:** Rosalind G. McAlpine, Matthew D. Sacchet, Otto Simonsson, Maisha Khan, Katarina Krajnovic, Larisa Morometescu, Sunjeev K. Kamboj

**Affiliations:** 1 Clinical Psychopharmacology Unit, Clinical, Educational and Health Psychology, University College London, London, UK; 2 Meditation Research Program, Department of Psychiatry, Massachusetts General Hospital, Harvard Medical School, Boston, USA; 3 Department of Neurobiology, Care Sciences and Society, Karolinska Institutet, Solna, Sweden

**Keywords:** Psychedelics, Psychedelic preparedness, Psychedelic therapy, Psilocybin, Meditation, Digital intervention, Person-centred, Qualitative, Co-design, Psychology, Health care

## Abstract

Psychedelic substances induce profound alterations in consciousness. Careful preparation is therefore essential to limit adverse reactions, enhance therapeutic benefits, and maintain user safety. This paper describes the development of a self-directed, digital intervention for psychedelic preparation. Drawing on elements from the UK Medical Research Council (MRC) framework for developing complex interventions, the design was informed by a four-factor model of psychedelic preparedness, using a person-centred approach. Our mixed-methods investigation consisted of two studies. The first involved interviews with 19 participants who had previously attended a ‘high-dose’ psilocybin retreat, systematically exploring their preparation behaviours and perspectives on the proposed intervention. The second study engaged 28 attendees of an ongoing psilocybin retreat in co-design workshops, refining the intervention protocol using insights from the initial interviews. The outcome is a co-produced 21-day digital course (Digital Intervention for Psychedelic Preparation (DIPP)), that is organised into four modules: Knowledge–Expectation, Psychophysical–Readiness, Safety–Planning, and Intention–Preparation. Fundamental components of the course include daily meditation practice, supplementary exercises tied to the weekly modules, and mood tracking. DIPP provides a comprehensive and scalable solution to enhance psychedelic preparedness, aligning with the broader shift towards digital mental health interventions.

## Introduction

Psychedelics are a distinct group of compounds recognised for their ability to induce profoundly altered states of consciousness. These substances are marked by pronounced alterations in sensory perception, mood, thought processes, perception of reality, and self-awareness^1–4^. Given the intensity of these effects, comprehensive psychological preparation is imperative to ensure the safety and well-being of individuals participating in clinical trials or engaging in therapeutic applications of these substances^5,6^. The significance of preparation becomes apparent when examining the millennia-long use of psychedelics in various Indigenous cultures^7–9^. These communities have not only recognised but also systematically implemented comprehensive strategies that integrate mental, physical, and spiritual dimensions in preparation for the psychedelic experience^10–13^. These holistic approaches are rooted in a deep understanding that navigating these altered states of consciousness without adequate preparation or guidance can pose significant risks and challenges^9,14,15^.

Consequently, the majority of contemporary clinical and experimental trials have included a specific clinician-led ‘preparation phase’, consisting of a diverse and non-standardised set of techniques, which are posited to significantly influence the course of the subsequent psychedelic experience^16,17^. This preparation is aimed at reducing the likelihood of psychologically distressing acute effects^18–20^ and extended difficulties following psychedelic drug use^15^, as well as increasing the likelihood of a potentially therapeutic experience^6,21–24^. Although there is a consensus in the current scientific discourse around the importance of adequate psychedelic preparation^6,25^, there is a pressing need for more rigorous evaluation of the various approaches, techniques and frameworks used to prepare participants^16^, as well as research specifically dedicated to developing instruments to optimise this process. As noted in a recent review by Thal et al.^17^, the current deficiency in available research means “it is impossible to draw definitive conclusions regarding the significance and impact of specific facets of preparatory sessions on therapeutic outcomes.” Advancing empirical research within this domain is therefore imperative to guide clinical best practices.

Despite the current ubiquity of *clinician-guided* preparatory sessions, the potential utility of self-directed preparation strategies to enhance individuals’ readiness for psychedelic experiences has not, to the best of our knowledge, been investigated. While a trusting therapeutic relationship seems to be a crucial prerequisite for psychedelic treatment^26^, extensive contact with a clinician in the preparation phase is resource-intensive and poses a significant challenge to the scalability and accessibility of psychedelic therapy^27^. By contrast, the use of digital technologies to deliver structured and standardised self-directed strategies allows for extensive, tailored and convenient psychedelic preparation outside the clinical setting^28^. Our prior work, which established a four-factor model of psychedelic preparedness^25^, suggested that a sizeable proportion of psychedelic preparation strategies could indeed be standardised and delivered remotely/digitally. These included strategies aimed at enhancing (i) individuals’ understanding of psychedelic substances and clarification of their expectations regarding potential immediate and long-term effects (Knowledge-Expectation), (ii) the extent to which individuals are mentally and physically ready to navigate the psychedelic experience (Psychophysical-Readiness), (iii) the introspective inquiry integral to a participant’s decision to engage with psychedelics (Intention-Preparation), and ensuring (iv) a safe and supportive environment in the post-psychedelic stage (Safety-Planning). Our previous work also implied that self-directed preparation strategies within each factor could be used flexibly to accommodate individual participant’ needs, preferences and pace, which would be guided by clinical judgement^29,30^.

As the healthcare sector increasingly integrates technology to enhance well-being and mental health, many services traditionally delivered face-to-face are being reimagined in the digital realm and have been shown to be non-inferior to more resource intensive in-person delivery^31,32^. As such, the use of web- and app-based technology, be it standalone or adjunctive, offers advantages for the delivery of self-directed psychedelic preparation interventions, such as improved accessibility, flexibility, anonymity, and cost-effectiveness^33–37^. Moreover, the use of digital interventions has shown to be pivotal in empowering participants in the management and delivery of therapeutic services^38–40^, by allowing them to take an active role in the management of their own health^30^. While recent initiatives have recognised the potential of digital tools in monitoring outcomes associated with psychedelic use^5^, there are no standardised digital interventions expressly designed for the preparation phase of psychedelic therapy. Addressing this gap offers a chance to harness digital interventions to make psychedelic therapies more efficient and convenient, reducing the burden on both participants and service providers.

While there is a growing body of evidence demonstrating the effectiveness of digital interventions in alleviating or preventing symptoms of mental health^27,41–44^, suboptimal user engagement poses a significant challenge in translating these benefits to real-world applications^37,45,46^. Recognising the need to promote better engagement with digital interventions, several studies have emphasised the necessity of taking a person-centric approach to digital intervention design^47–51^. The rationale for involving relevant stakeholders from the start, and indeed working closely with them throughout, is that they can help to identify priorities, understand challenges, and find solutions that enhance the likelihood of successful implementation^52–57^. Although recent guidelines in psychedelic science^58^ have advocated for a person-centric approach and have emphasised the inclusion of patients and the public more broadly to enhance the credibility and impact of research while promoting accountability, transparency, and relevance^59^, the inclusion of such perspectives is still noticeably lacking in the field^60^.

In light of the recognised potential of digital and remotely-delivered psychosocial interventions within psychedelic science and therapy^5,61–63^, the purpose of this research was to develop and refine a self-directed Digital Intervention for Psychedelic Preparation (DIPP) using a person-centred, mixed-methods approach. Aligned with our recent four-factor model of psychedelic preparedness^25^, DIPP’s content and structure were developed through a phased process involving two studies:

- Study 1—*Initial Intervention Development* This phase of the project aimed to identify essential components for psychedelic preparation by conducting in-depth interviews with psilocybin retreat participants. The insights obtained were crucial in determining the foundational elements and thematic structure of DIPP, ensuring the intervention’s relevance to actual user experiences.
- Study 2—*Intervention Component Refinement* Building upon the findings from Study 1, this phase involved iterative feedback through co-design workshops with psilocybin retreat attendees. This step was key to refining DIPP’s components, enhancing its user-centeredness and implementability across varied contexts.

While DIPP has been tailored to be suitable for both clinical and retreat contexts (and potentially adaptable for non-therapeutically-oriented research studies), our data collection efforts focused on psilocybin retreat participants. This population was selected as they were more readily accessible for the purposes of this research. The insights derived, however, are expected to be translatable across other clinical and non-clinical settings.

Our intervention was structured following the UK Medical Research Council (MRC) framework for complex interventions^52,57,64^ which delineates four stages: Development, Feasibility and Pilot Testing, Evaluation, and Implementation. Within this study, we concentrated on the ‘Development’ stage, prioritising the early assessment of feasibility and acceptability through qualitative methods, as recommended by the MRC^65–67^. The description of our intervention follows the ‘Template for Intervention Description and Replication’ (TIDieR) checklist^68^ and the GUIDance for the rEporting of Intervention Development (GUIDED) framework^69^ (Supplementary Material 3A/B).

## Study 1

### Methods

All research was performed in accordance with the Declaration of Helsinki and all procedures were reviewed by, and received approval from, the University College London Research Ethics Committee (9437/001). All studies were performed in line with relevant guidelines and regulations^70–75^.

#### Recruitment

Participants were recruited through collaborating psilocybin retreat centres. While there are some differences in the structure and content of each retreat centre’s program, the centres were selected to be as similar as possible in program structure and types of support offered. For additional contextual information on collaborating psilocybin retreat centres, see Supplementary Material 1A.

Information about the study was disseminated to potential participants by email, outlining the study’s aims and containing a hyperlink to an information sheet detailing the study protocol. The inclusion criteria specified that participants must be over the age of 18, have experienced a ‘significant change’ as a result of a psilocybin retreat, and be proficient in English. Informed consent was acquired electronically, verifying each participant’s understanding and agreement to the study’s terms. Nineteen participants met inclusion criteria and completed the study.

#### Procedures

Participants (n = 19) underwent a semi-structured interview administered by the lead author (RM) via video conferencing (Microsoft Teams). These interviews took place on average 3 months post-retreat (Range 1–9 months).

The interview schedule had three distinct parts to comprehensively explore the various dimensions of participant preparation. Part A enquired about participants’ preparatory activities and the perceived outcomes resulting from these actions. Part B focused on their perceptions of the effectiveness of specific preparation techniques, discussion of difficulties encountered during the preparation process, and ideas for potential areas where additional instructions might benefit the experience. Part C elicited participants’ perspectives regarding a proposed preparatory intervention. They were asked to appraise its potential utility, propose essential features and components, offer guidance on elements to exclude based on personal experiences, and express their preferences regarding the mode of delivery and optimal duration for the program.

#### Data analysis

##### Qualitative responses

Thematic analysis^70,71^ was conducted using qualitative analysis software NVivo 14 to identify key methods and outcomes related to participants’ preparatory experiences. Recorded interviews were transcribed using Microsoft Teams’ live transcription feature, and all transcriptions were cross-checked for accuracy against the original recordings. Collaborative coding, combining inductive and deductive approaches, was conducted by RM, MK, and MM.

**Figure 1:**
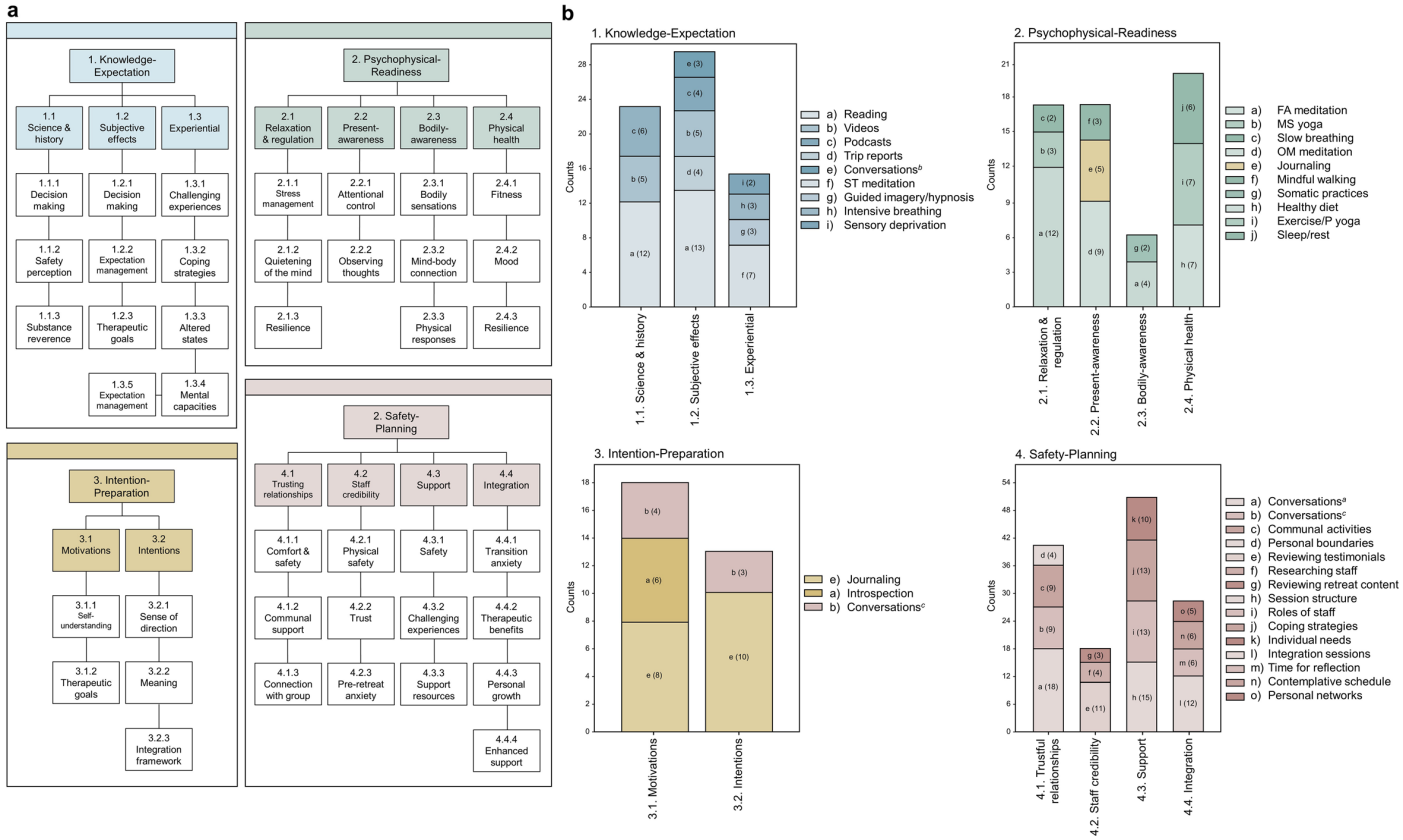
Thematic structure and corresponding activities. Panel A delineates the four main themes and their corresponding subthemes. Panel B presents bar charts illustrating the activities associated with each theme/subtheme.

The theoretical framework guiding this analysis was based on the four-factor model of ‘psychedelic preparedness’ developed in our previous paper^25^. The four factors/themes consisted of (i) Knowledge-Expectation (KE), (ii) Intention-Preparation (IP), (iii) Psychophysical-Readiness (PR) and (iv) Safety-Planning (SP). Notably, the occurrence of a preparatory activity within a particular theme or subtheme was determined by its unique role as identified by participants, resulting in some activities being associated with multiple themes. For instance, the activity ‘Journaling’ falls under both PR subtheme 2.2 (‘Present-moment awareness’), and IP subtheme 3.2 (‘Intentions’). The thematic structure and the corresponding activities for each subtheme is illustrated in Fig. 1**.**

Meditation and yoga practices were commonly reported across various subthemes. Previous research has classified these practices according to their primary objectives and methodologies, giving rise to various proposed typologies^76–80^. While acknowledging their limitations, we adopted gross categorisation systems for both meditation and yoga practices. For meditation, we used the categories of focused-attention, open-monitoring, compassion and loving-kindness, and self-transcendence^81^. For yoga, we employed power and meditative-stretch taxonomy^82,83^.

##### Descriptive statistics

Demographic data was collected in numerical or categorical forms. The qualitative data underwent Structured Tabular Thematic Analysis (ST-TA)^84^, a process that converts qualitative responses into numeric codes to facilitate a frequency and percentage distribution analysis of different themes, thereby creating a quantitative summary of the data. The data were analysed using Python (Version 3.12).

### Results

#### Demographic

Table 1 shows the demographics and psychedelic use variables for the 19 participants who completed this study.

**Table 1:** Sample (n = 19) demographic and psychedelic use variables.

Variables	M ± SD	Range
Age (years)	42.37 ± 12.37	37–77
Months since retreat	3.16 ± 2.09	1–9
Days spent preparing for retreat	17.68 ± 11.29	3–40
Minutes per day spent preparing for retreat	37.63 ± 18.06	10–60

	n	%
Gender		
Male	7	36.84
Female	12	63.16
Education		
High school/college	1	5.26
Undergraduate	8	42.11
Postgraduate	10	52.63
Ethnicity		
White	14	73.68
Black	1	5.26
Latino/Hispanic	1	5.26
Mixed	3	15.79
Religion		
Not religious	14	73.68
Christian	5	26.32
Lifetime psychedelic use		
Never before	12	63.16
On 1 occasion only	4	21.05
On 2–5 occasions only	3	15.79
Location of psilocybin retreat		
Mexico	12	63.16
Netherlands	7	36.84

#### Part A: identifying preparatory activity themes

We deductively identified four main themes in participants’ description of preparatory activities, based on the four-factor model of ‘psychedelic preparedness’ developed in our previous paper^25^. Each of these themes had its own set of related subthemes, which were identified inductively. A visual representation of this thematic structure, along with the activities connected to each subtheme, can be found in Fig. 1. This section exhaustively describes the derivation of the full range of subthemes. For readers more interested in the narrower set of ‘most effective’ strategies identified by participants which formed the basis for components of DIPP, these can be found under section heading Part B’ below.

#### Theme 1: knowledge-expectation

The Knowledge-Expectation theme refers to activities that participants described as undertaking in order to improve their understanding of psychedelic substances and their resultant expectations regarding the potential immediate and long-term effects.

##### Subtheme 1.1: acquiring knowledge about the science and history of psychedelics

Participants demonstrated a robust commitment to learning about the scientific foundation and historical background of psychedelic substances. Twelve participants (n = 12) reported proactively seeking scientific literature, books, and online materials, while others made use of expert-led videos (n = 5) and podcasts (n = 6) as educational resources.

This self-directed education allowed participants to make more **informed decisions about psychedelic use (1.1.1)**. By improving their understanding of psychedelics, participants felt better equipped to assess their readiness for, and the appropriateness of the psychedelic experience. A solid foundation of knowledge also enabled the appraisal of potential risks, thereby addressing and clarifying initial uncertainties.

[Doing my own research] helped me [to] weigh out the pros and cons, before deciding whether this was something I wanted to do. (P5)

The more I learned about [psilocybin] […] the more I felt that my decision to go on this journey was a sensible thing to do. (P14)

Expanded knowledge also influenced participants’ **perception of the safety of psychedelics (1.1.2)**. Participants who actively sought information on both the historical use and scientific understanding of these substances typically perceived psychedelics as safer than they initially believed.

I [discovered] that cultures have been using these substances for centuries […] It eased my concerns. It wasn’t just a recent fad. (P6)

[This knowledge] definitely made me feel more comfortable about trying them myself […] they are literally giving them to people in dozens of [universities] around the world… (P12)

Self-initiated education also resulted in a notable change in **participants’ attitudes towards psychedelics (1.1.3).** As participants acquired knowledge on the historical, cultural, and scientific facets of these substances, initial neutrality or curiosity often evolved into variations of “respect” (P4). This change frequently coincided with a decrease in initial “misconceptions” (P7) and a more informed, positive perception of psychedelics.

[…] opened my eyes to how powerful these substances can be […]. There’s a depth to [psilocybin] that demands respect. (P4)

It made me rethink everything. (P18)

It was like separating fact from fiction, which made me feel way less anxious about it all. (P7)

##### Subtheme 1.2: acquiring information about subjective psychedelic effects

Participants leveraged multiple strategies to gain insights into the potential subjective effects of psychedelic experiences. Thirteen participants (n = 13) consulted scholarly articles, books, and blogs authored by experts in the field. Four participants (n = 4) found value in personal trip reports, often available on platforms such as Erowid and Reddit, while others explored multimedia resources such as videos (n = 5) and podcasts (n = 4) featuring personal accounts of psychedelic experiences. A smaller subset of participants (n = 3) directly sought conversations with experienced peers to gain firsthand insights.

These activities greatly facilitated the **decision-making processes (1.2.1)** of participants. By understanding the potential spectrum of experiences and assimilating personal accounts, participants were empowered to make more “informed” (P9) decisions about the appropriateness of such experiences for their current situation.

[Knowing what to expect] helped me to make a […] decision about whether this was something for me, right now. (P16)

Honestly after hearing from some of these patients I was a little scared, [but] I realised [that] this was basically something I needed to do too... I [wasn’t] expecting to see rainbows and fairies and I didn’t even want to. I just want to heal. (P9)

Information gathering also assisted in **setting realistic expectations for their own psychedelic journeys (1.2.2).** The importance of gaining clarity on the potential experiences was emphasised, with the goal of mitigating any “surprise or shock” (P13). This understanding led participants to approach the experience with a better sense of preparedness and a realistic mindset.

[…] helped me set expectations that were grounded in reality, not just some abstract idea of what I thought it might be like. (P11)

... set me up to manage whatever came my way. (P19)

The assimilated information prompted participants to **reflect on their therapeutic goals (1.2.3).** Assessing potential subjective effects allowed several participants to delineate anticipated outcomes from the psychedelic journey, creating a “roadmap” (P3) of desired milestones. This process fostered a deliberate consideration of personal therapeutic advantages, focusing on the tangible benefits of the psychedelic experience.

I guess understanding what could [potentially] happen on these trips really made me consider my own [goals]. (P17)

[...] I was focusing on what I wanted to achieve from the whole thing. [It] kind of gave me a roadmap. (P3)

##### Subtheme 1.3: undertaking experiential mind–body exercises to familiarise with potential psychedelic phenomena

Participants highlighted the role of experiential exercises in preparing for forthcoming psychedelic experiences. Some participants partook in various practices to acquaint themselves with possible psychedelic phenomena, including self-transcendence meditation techniques such as non-dual and transcendental meditation (n = 7), guided imagery and hypnosis (n = 3), intensive breathing exercises like Holotropic Breathwork and the Wim Hof Method (n = 3), and sensory deprivation activities such as floatation tanks (n = 2).

Participants noted that these practices markedly **increased their confidence in navigating the psychedelic journey (1.3.1)**. Techniques such as self-transcendence meditation and Holotropic Breathwork provided an acquaintance with the potential variability of the psychedelic experience. Therefore, initial exposure to an altered state of consciousness via these preparatory exercises appeared to improve participants’ confidence in navigating the unfamiliar psychedelic experience.

Going through all that really helped me get cosy with the unpredictable. (P9)

[Meditation] gave me confidence to navigate whatever came up. (P10)

[...] my [training in Vedanta] gave me a bit of a warm up… I already knew what it felt like to really let go. (P11)

These activities furthered the cultivation of **adaptive coping strategies for the potential challenges of the psychedelic journey (1.3.2).** They offered a “safe space” (P6) for participants to navigate difficult situations, practise ‘surrendering’, and refine their capacity to ‘let go’. Methods like using affirming mantras (e.g., “I am not my thoughts”) and calming breathwork (e.g., pursed lip breathing, 4–6-8 breathing) were applied to address anxiety during these experiential exercises. Consequently, participants were better prepared to handle emotionally intense moments during the actual psychedelic experience.

[The visualisations] let me run through all sorts of scary ‘what ifs’ without actually being out of my head. (P2)

I was kind of learning to let go, to surrender, to peel back layers. (P15)

Experiential exercises were also noted to foster **familiarity with altered states of consciousness (1.3.3)**, giving participants a more nuanced understanding of potential shifts in perception and awareness during the psychedelic experience. For example, one participant expressed that they had experienced an “intricate tapestry of consciousness” (P15), enabling them to approach their psychedelic experiences with insight, openness, and readiness, rather than apprehension.

At first, [the effects of holotropic breathwork] were just intense and physical, but then it sort of broke open into something more [...] I started seeing patterns, feeling connected to things outside myself. (P1)

[...] it’s like I stumbled onto a whole new side of my mind. (P3)

[During this meditation] you’re awake, but it’s not your everyday kind of awake. It’s like being in this peaceful bubble where your mind can wander off into different directions […] that really helped me get a [sense] of what being on [psilocybin] might feel like. (P6)

**Increased self-awareness (1.3.4)** was another notable outcome of these experiential exercises. Participants reported that the practices fostered a deeper understanding of their mental processes and patterns, enabling them to approach their psychedelic journeys with a more metacognitive stance.

It showed me just how much control and insight I could actually have. (P3)

I started realising that my mind wasn’t just something that things happened to. (P7)

Finally, these preparatory exercises were found to be useful in **managing expectations effectively (1.3.5).** Through such exercises, participants gained insights into the potential for profound revelations, emotional challenges, and unexpected experiences during the psychedelic journey. For several participants, this helped them to recalibrate and align their expectations to the depth and intensity of the forthcoming experiences.

[...] it gave me an idea that the actual experience could bring […] challenging moments.” (P15)

“[Breathwork] kind of hinted at the stuff that might come up. (P15)

#### Theme 2: psychophysical-readiness

The Psychophysical-Readiness theme describes how participants prepared themselves mentally and physically for navigating the complexities of the psychedelic experience. It encompasses the readiness to confront the emotional or physical phenomena that may arise, acceptance of potentially uncomfortable aspects of the experience, and a trust in one’s mind and body to manage the journey safely.

##### Subtheme 2.1: promotion of a relaxed and regulated psychophysical state

Participants utilised various practices to cultivate a relaxed and regulated psychophysical state in preparation for psychedelic experiences. These activities comprised focused-attention meditation, such as breath awareness (n = 12). A fraction of participants also practised meditative-stretch yoga, such as Hatha Yoga (n = 3). Some participants (n = 2) engaged in slow, deep breathing exercises such as ‘4–6 breathing’ and ‘box breathing’, known for inducing relaxation.

Participants identified these practices as beneficial for **stress management (2.1.1)**, regulating their mental and physical well-being, and fostering calmness during challenging situations. Hence, the practice of these techniques, particularly at the start or end of the day, was associated with significant reductions stress and emotional regulation.

I found a few moments every day, especially when I felt overwhelmed or needed a break […] I would sit down, shut my eyes, slow down my breathing, and try to centre myself to relax and regain balance. (P2)

The idea was to […] try to step away from the [stresses] of my life, creating a sense inside that everything was okay and still. (P7)

[Deep diaphragmatic breathing] allowed me to tap into a sense of slow, grounded stability, even during moments of real [stress]. (P2)

[Hatha yoga] bec[a]me my go-to escape from the craziness of life. (P2)

Many participants also emphasised that these practices led to a **quieting of the mind (2.1.2).** They described discovering a sense of stillness, slowing down the pace of life, and a reduction in internal dialogue through their engagement in these activities.

[I] strived to carve out a period where I could experience calmness, [quietening] the ‘monkey mind’. (P1)

[...] that voice in my head would go away, even if just for a few moments, and I’d just be flowing and moving and breathing without any distractions or voices. (P19)

My main goal was to quiet my mind and find a sense of inner calm. (P3)

[Mindful meditation] helped me to slow down my mind and relax. (P16)

When I was in [yoga] flow, that voice in my head would go away, even if just for a few moments […] and I’d just be flowing and moving and breathing without any distractions or voices. (P19)

Through sustained training or extended practice, several participants reported an enhancement in **personal resilience (2.1.3)**, with an increased ability to adapt to and manage challenges as they advanced in their preparation for psychedelic experiences.

I basically build up the mental strength to be able to do it […] By practising staying calm and focused. (P7)

At first I was bored but as I kind of got better at the whole thing it started to get way more eas[ier]. (P3)

[...] my mind was like a hamster wheel, constantly racing with thoughts, [but] with consistent [meditation] practice I [am now able] to effortlessly enter a still state.” (P15)

“I was pretty disciplined [about yoga] because I wanted to train my mind and body to be strong and ready. (P4)

##### Subtheme 2.2: cultivating present-moment awareness

Participants reported engaging in various practices to cultivate present-moment awareness as part of their preparation for psychedelic experiences. These activities included open-monitoring meditation (e.g., mindfulness, Vipassana) (n = 9). Participants also engaged in reflective writing exercises (n = 5) and mindful walking (n = 3), often in nature.

Participants engaging in these practices reported noticeable enhancements in **attentional regulation and sustained focus (2.2.1).** For instance, the implementation of these techniques often resulted in an increased capacity to direct cognitive resources effectively and reduced the impact of intrusive thoughts. In the case of reflective writing, for instance, the slow and deliberative nature of the task requires a high level of patience and focused attention. Participants reported that this particular practice compelled them to stay closely attuned to their thoughts, thereby fostering an enriched engagement with their internal cognitive landscape.

Writing all those thoughts down really forces you to be patient and stay with your thoughts because you can only write as fast as you can. I had to really focus on what I was thinking and allow that to come out through the pen. (P2)

[FM meditation] made me less distracted, like I wasn’t jumping around with thoughts. (P6)

[...] I would pay attention and focus on every single step and every single tree […] I could focus on everything way more clearly. (P16)

I felt more present [after FM meditation]. I would be having a conversation with my partner and I was actually there. I wasn’t elsewhere in my mind. I was there with them, *in* that moment, focusing on what they were saying and how we were sharing words. (P13)

These practices also fostered an increased capacity for participants to **observe their thoughts (2.2.2).** Techniques such as open-monitoring meditation reportedly facilitated participants’ ability to decenter from their thoughts, perceive them with objectivity, and refrain from automatic judgement or evaluation. This promoted a more mindful, nonreactive relationship with their ongoing thought processes, resulting in what one participant characterised as a “healthier relationship with [their] own stream of consciousness.” (P8).

It was like a little peg just dropping the thoughts off in one room and letting me move onto the next. (P4)

By writing all of this stuff down, I would then just have it there, in a little packaged form, and then I could shut the book and be like okay yeah that’s what I felt but that’s just it [...] They are just words, they are just feelings, and I could quite literally just look at them… (P2)

[...] I just could way more easily see the distractions come up and let them go. (P16)

[…] and so the more I was able to learn to just listen to those thoughts and just watch them, I realised they were normal and they eventually just, kind of, went away. (P9)

##### Subtheme 2.3: cultivating awareness of body sensations

Participants utilised specific practices to enhance their awareness of bodily sensations in preparation for psychedelic experiences. Body scan meditation (e.g., FA) was a relatively common practice among participants (n = 4). Some participants also engaged in specific somatic or movement practices , namely Feldenkrais Method (n = 1) and Alexander Technique (n = 1), to further cultivate this awareness.

Several participants reported a refined ability to **perceive internal physical sensations (2.3.1)** through these types of practices. This heightened awareness ranged from recognising the subtleties of their heartbeat, as one individual became acutely attuned to variations in rhythm and intensity, to sensing delicate shifts in posture and the gentle rise and fall of their breath. These experiences were described as contributing to an enriched sense of presence and connection with the physical body, both within daily life and throughout the psychedelic journey.

[...] I would just get more and more aware of my own heart and the way it was beating. (P6)

I started noticing the gentle rise and fall of my breath, the slight tingling in my fingertips, and… the [sensations of] warmth or coolness in different parts of my body. (P9)

I could sense the […] subtle shifts in my posture. (P12)

I was able to notice tension… and areas of discomfort, and with gentle adjustments, I learned to release and realign my body. (P11)

The practice of these techniques also fostered a more nuanced understanding of the **mind–body connection (2.3.2)**. Participants were able to recognise and interpret the intricate relationship between physical sensations and psychological states. For example, some noted how physical tension in specific areas such as shoulders and jaw could correspond to feelings of stress or anxiety. Others reported that focusing attention on different parts of the body would evoke memories and feelings, signifying a deep, interconnected relationship between the physical and emotional self.

I [noticed that] when I was feeling stressed or anxious, my body would tense up, especially in my shoulders and jaw. (P10)

I learned to recognise how my body responded to different emotions. (P14)

[...] as I moved my attention to different parts of my body, different things would come up, like memories and feelings. (P6)

Finally, participants found that these practices equipped them with improved control over their **physical responses to stress (2.3.3).** They described gaining proficiency in regulating their nervous systems and making deliberate decisions about bodily reactions. Some expressed that this practice allowed them to notice tension or discomfort, then consciously release and realign the body. This enhanced control contributed to a more intentional and “empowered” (P6) engagement with their bodies, which was perceived to enrich their overall psychedelic experiences.

[...] I got better at regulating […] my nervous system.. (P9)

It was cool because [practising the Alexander Technique] helped me to learn how much effort I needed to move, and I could look inside and make deliberate decisions about when and how I was going to react to things and move my body. (P12)

I could catch myself in the moment and consciously release the tension. (P10)

##### Subtheme 2.4: optimising overall physical health

Participants adopted various strategies aimed at nurturing their bodies and enhancing overall health and well-being in anticipation of their psychedelic experiences. These strategies included adopting a healthy and balanced diet (n = 7), engaging in regular exercise, including cardio, strength training, and power yoga (n = 7), and prioritising sufficient sleep and rest (n = 6).

Participants observed **improved fitness (2.4.1)**, including weight loss and increased cardiovascular endurance, following consistent commitment to regular exercise and healthy eating habits. Rather than aiming to achieve an idealised state of physical health, participants sought a sense of feeling strong, healthy, and prepared for their upcoming experiences. This level of physical readiness served to instil confidence and contribute to an overall sense of wellbeing.

I actually got really fit, maybe the most fit I’ve been in years. (P12)

[My body] felt good and way lighter… I had so much more energy and actually wanted to move. (P11)

It wasn’t about becoming some kind of health guru… It was about feeling like my body was in good shape […] strong and healthy, and kind of cleaned out, ready. (P16)

Participants also reported an improvement in their **baseline mood and wellbeing (2.4.2)**, resulting from the combined effect of regular exercise, balanced nutrition, sufficient sleep, and self-care ‘rituals’. The adoption of these healthy lifestyle habits led to an increase in energy levels and positive feelings, creating an optimal emotional “foundation” (P5) for their forthcoming psychedelic experiences.

Cutting out all that crap [food] just made me feel good. (P18)

[Regular yoga] makes me feel so much better… so much more full of energy and positivity. (P14)

... when I started [prioritising sleep] I felt way less anxious. (P8)

Several participants found that regular engagement in physically challenging activities such as power yoga contributed to an increased **resilience to physical discomfort (4.2.3)**. The discipline cultivated during these rigorous practices was perceived to carry over into their psychedelic experiences, allowing them to manage and navigate any arising bodily sensations more effectively.

[...] they help me to… keep going even when it feels like I can’t go on […] because I know I can do it, and I know I’ll be fine. (P14)

It was a lot about discipline and pushing through. (P4)

#### Theme 3: intention-preparation

The Intention–Preparation theme summarises the introspective journey that underlies participants’ decision to use psychedelics. This process involves thoughtful deliberation on the motivations behind the psychedelic use, such as self-exploration, therapeutic purposes, or spiritual growth. Integral to this theme is the establishment of setting clear intentions for the experience, providing a sense of direction and purpose.

##### Subtheme 3.1: identifying motivations for using psychedelics

Participants engaged in reflective processes to understand their motivations for using psychedelic substances. These processes included journaling (n = 8), introspection (n = 6), and discussing with friends or psychedelic communities (n = 4).

The process of reflecting on their motivations led participants to an increased **self-understanding (3.1.1)**. Participants reported gaining clarity regarding their personal characteristics, life circumstances, preferences, and dislikes. For instance, journaling about their reasons for using psychedelics facilitated an in-depth self-evaluation, leading to a comprehensive understanding of their personal attributes and behaviours.

[Journalling] made me really think about all of the parts I have to myself… (P15)

I had to admit… that I wasn’t this confident person I [had] made [myself] out to be. (P14)

Upon introspection of their motivations, participants found themselves better aligned with their **therapeutic goals (3.1.2)**. The act of contemplating the potential benefits of psychedelic use in relation to their current life situations aided them in identifying and clarifying the specific outcomes they intended to achieve from their psychedelic experiences.

[...] you start to notice, or like [to] identify, which bits of your story you want to let go of. (P3)

[...] when I really thought about why I was going, it was way more dense than I first realised… and it helped me work out really what I wanted to make of this retreat. (P16)

##### Subtheme 3.2: establishing intentions for the experience

Participants frequently spoke about explicitly ‘setting intentions’ for their psychedelic experiences. This was typically accomplished through journaling (n = 10), meditation (n = 3), and engaging in discussions with friends or psychedelic communities (n = 3).

Setting clear intentions provided a heightened **sense of direction for the psychedelic experience (3.2.1).** These pre-established intentions acted as a guide or “anchor” (P14), helping maintain focus amidst the complex sensory and cognitive alterations induced by psychedelics.

[Journalling] gave me a clear sense of direction for my experience. (P9)

It was like drawing a map for my journey. (P18)

[Speaking with others] gave me a better understanding of what I wanted from my experience. (P17)

The establishment of explicit intentions also augmented the **meaningfulness of the psychedelic journey (3.2.2)**. “Meaningfulness” was defined by participants as the personal relevance and emotional resonance of the experience. They expressed that having distinct, personal objectives before the psychedelic experience increased the perceived value and significance of the insights gained during the journey.

[Journalling] infused my experience with a deeper sense of purpose and meaning. (P14)

[Mindful meditation] allowed me to feel the significance of it all. (P16)

[Meditation] helped me to get super mindful of my intention, which helped me connect with it on a deeper level. (P1)

[Engaging in discussions] added depth to the [psilocybin] journey. (P6)

The presence of defined intentions offered a **practical framework for integration (3.2.3)** following the psychedelic experience. Participants referenced their set intentions as guiding principles or “touchstones” (P12) for interpreting and incorporating their psychedelic insights into their everyday lives.

It was interesting to see how my intentions changed and evolved as we got closer to the retreat and also during it… I still sometimes look back at [them] now and they really remind me of how far I’ve come. (P7)

I could reflect back on my initial goals and see how they’ve manifested. (P9)

#### Theme 4 safety–planning

The Support-Planning theme explores the importance of ensuring a safe and supportive environment during the psychedelic experience and the need for post-experience planning. This theme focuses on establishing a trustful connection with those present during the experience, ensuring the safety of the substance, preparing friends and family for potential changes, and making a contingency plan for possible challenging moments during and after the experience.

##### Subtheme 4.1: cultivating trustful relationships

Participants prepared for their psilocybin sessions by establishing trust with those in attendance, accomplished via dialogues with facilitators or guides (n = 18), preliminary conversations with peers (n = 6), and participation in communal activities such as breathwork, meditation, and ecstatic dance (n = 9). The importance of clear communication and respect for personal boundaries during the psychedelic session was also emphasised (n = 4).

The establishment of trustful relationships **enhanced participants’ sense of comfort and safety (4.1.1)**, resulting from both pre-retreat interactions and mutual respect for individual boundaries. Trust was fostered through initial discussions with facilitators and peers prior to the retreat, which served to “humanise” (P3) the forthcoming experience and cultivate a sense of familiarity. In addition, mutual recognition of and respect for personal space contributed to an environment of trust and safety.

Getting the chance to chat with [the facilitators/guides] before the retreat was really helpful […] [It was] relieving to talk to an actual person. (P13)

Speaking to [the facilitators/guides] definitely put some of my nerves to rest. (P6)

I was able to feel more comfortable around [the other guests] because I had already met them on Zoom. (P14)

I think we all could feel it… like safe in this container, trusting that everyone would basically stick to what we signed up for and respect those boundaries. (P3)

The development of relationships with fellow participants and retreat personnel enabled the **growth of a strong communal support network (4.1.2)**. This experience was often described by participants as joining a new familial structure, underpinned by reciprocal watchfulness. The reassurance derived from the presence of others, coupled with the knowledge of mutual safeguarding within the group, engendered a sense of ease, underscoring the essential role of a supportive community in the participants’ experience.

[...] like becoming a member of a new family, with people looking out for me. (P7)

It was reassuring to know that I was not alone in my journey. (P1)

[...] made me feel so at ease knowing we had each other’s backs. (P15)

These relationships also significantly contributed to **promoting a sense of unity within the group (4.1.3)**. Emphasis was placed on collective experiences such as workshops and sharing circles, which swiftly catalysed the formation of close bonds among participants. This rapid development of connection often took participants by surprise. Such shared experiences proved critical in amplifying group coherence and fostering an environment conducive to empathy and support.

I was amazed by how tight-knit our group became in such a short span of time. (P10)

Doing the [morning workshops] together definitely made us feel closer. (P11)

We began to form bonds early on. (P12)

##### Subtheme 4.2: verifying retreat and staff credibility

Participants engaged in thorough online research to assess the credibility of the retreat and its staff. This process included examining past participant reviews and testimonials (n = 11), investigating the experience, qualifications, and skills of the retreat staff (n = 4), and assessing the retreat’s digital footprint, including social media presence and website content (n = 3).

Rigorous scrutiny of retreat and staff credibility significantly bolstered participants’ **perception of physical safety (4.2.1)**. An abundance of positive reviews and testimonials often solidified confidence in the legitimacy of the retreats. A sense of relief was noted among participants, attributable to the realisation that the staff were proficiently equipped with specialised skills and comprehensive experience, thereby fostering an environment of security.

They had tons of reviews […] you could tell straight away it was legit[imate] (P11)

[...] everyone seemed to have a special skill […] [It] felt like I was in safe hands.”(P3)

Complementing the enhanced sense of safety, this verification process precipitated a **heightened level of trust in the retreat staff (4.2.2)**. The dedication, expertise, and professionalism exhibited by the staff through their digital platforms were pivotal in nurturing this trust. Further trust development was facilitated by the staff’s responsive engagement with inquiries on social media, demonstrating their expertise and commitment to the retreat.

Seeing their dedication, their depth of knowledge...it was so reassuring, you know. I felt...I felt secure. (P10)

[The retreat staff] weren’t just randomly picked people... they were experienced, skilled...[I] felt I was stepping into a well-run, professional space. (P18)

Seeing them answer questions and interact with their followers [on social media]... I could tell they knew what they were talking about. (P7)

I watched some of their live sessions...their way of explaining and guiding through the process...it was really impressive. (P14)

Subsequently, the procedure of validating retreat and staff credibility effectively **alleviated pre-retreat anxiety (4.2.3)**. Online testimonials and reviews depicting transformative experiences of former participants fostered a sense of calmness among prospective attendees. The resultant trust, founded on the retreat staff’s proven experience managing similar retreats and handling diverse situations, was a considerable factor in minimising participants’ anxiety.

...when you read about other people having these [transformations] it makes you feel a lot calmer about the whole thing. (P19)

[I trusted them] and it took a bit of weight off… because I knew how many times they had done this and how they had seen absolutely everything. (P5)

##### Subtheme 4.3: preparing in-experience support

Participants prepared for the in-experience support during the psilocybin session by acquiring a comprehensive understanding of the session’s structure (n = 15) and the roles of the retreat staff during the session (n = 13). They also focused on the development of personal coping strategies (n = 13) and actively communicated their individual support needs and preferences to the retreat facilitators (n = 10).

A comprehensive understanding of the session’s structure and the roles of the retreat staff significantly **enhanced the participants’ sense of safety (4.3.1).** Workshops delineating the ceremony and open Q&A sessions provided valuable clarity and alleviated uncertainties. Additionally, personal consultations with facilitators about individual support needs further instilled a sense of calm among participants. The development of personal coping strategies, such as grounding and mindfulness techniques, also served as a comforting resource.

[The staff] spent about two hours doing a ‘what is ceremony’ workshop, where they explained what will happen during the [psilocybin session] [...] At the end they opened it up to questions [...] Before this meeting, I didn’t really know what to expect, so it was a relief to have all my questions answered. (P2)

I had a long chat with one of the facilitators about my [support needs] on the second day of the retreat [...] [which] made me feel a bit calmer about the [psilocybin session] [...] I knew they were there to help and guide me. (P8)

“It was comforting to know I could come back to my breath if things started to get difficult...” (P17)

This preparation process also expanded the participants’ **capacity to manage challenging psychedelic experiences (4.3.2)**. Participants reported that guidance from facilitators, such as advice on navigating difficult moments during the ceremony, proved “transformative” (P9). Additionally, reassurances from the staff about their continual presence and watchfulness enabled participants to let go and fully engage in the experience. Participants also recalled utilising grounding techniques taught by facilitators to effectively manage intense moments during the session.

[...] when I [faced a difficult moment] during the ceremony, I remembered the advice from [one of the facilitators] to ask the question: “is this medicine, if not you must go” [...] It changed the whole scene. (P3)

[The staff] reassured me that they would keep an eye on me, and that acknowledgment… that feeling of being held… really allowed me to let go during the ceremony. (P14)

During a particularly intense moment in the ceremony, I felt a surge of anxiety [...] I remembered one of the [strategies] I had been told by [a facilitator]. I visualised myself as a tree, with the roots spreading deep into the earth, grounding me literally into the floor, making me immovable [...] I concentrated on my breath, imagining each exhale sending my roots deeper and deeper into the ground. The anxiety didn’t disappear completely, but it became way more manageable... (P10)

Finally, preparing and understanding in-experience support in advance of the psilocybin sessions also **optimised the use of such support resources (4.3.3)**. Participants felt empowered to actively seek help when needed, as they were well-informed about how to communicate their needs during the session. Facilitators’ timely and empathetic responses to these requests further facilitated participants’ navigation of the experience. This process, in turn, led to an optimised use of the support resources available to the participants.

... during the second ceremony I *knew* I could put my hand up and ask for some help… [A facilitator] came to me and sat by me. [They] asked if I wanted a hand… They stroked my head and helped me to slow down my breathing… and that was exactly what I needed… (P2)

... I asked how I should let the facilitators know if I wanted a hand hold. (P8)

##### Subtheme 4.4: preparing a post-experience integration plan

Participants prepared for their post-experience integration by arranging ‘integration’ sessions with retreat staff to discuss and interpret their experiences (n = 12). Additionally, they allocated adequate recovery and reflection time following the retreat (n = 6) and created a consistent schedule for integration or contemplative practices (n = 6). Participants also proactively communicated with their personal networks about potential changes following the retreat (n = 5).

Efforts towards preparing a post-experience integration plan significantly **reduced participants’ post-retreat transition anxiety (4.4.1)**. Participants reported that taking time off after the retreat eased their transition back into their daily routines, making the process less abrupt and more manageable. Planning to establish regular practices, such as daily meditation, even for short periods, was recognised as a significant aid in transitioning back into everyday life.

After the retreat, I [took] a week off just to unwind [...] It eased my re-entry into my daily routine, making it less abrupt and more... gentle. (P16)

Establishing a morning meditation routine [after the retreat]... even if it was only five minutes a day [...] really helped me transition back into my everyday life. (P11)

These planned post-retreat arrangements also led to **continued therapeutic benefits (4.4.2).** Participants found that integration sessions with retreat facilitators enabled them to process their experiences more effectively and apply the lessons learned during the retreat to their daily life and relationships. These sessions, combined with consistent meditation and self-reflection practices, were seen as effective strategies to tap back into the therapeutic “mushroomy” (P10) feeling and handle daily life triggers.

After the retreat, [meditation] became a way for me to tap back into that ‘mushroomy’ feeling [...] It was like a daily mood boost. (P10)

[The integration sessions with the retreat facilitators] helped me to understand and process the tougher parts of my experience [...] They helped me find ways to actually make changes and apply some of those lessons to my life and relationships. (P7)

The retreat was just the beginning. As I integrated more meditation and self-reflection into my life [...] I noticed the lessons from the retreat were helping me to handle triggers in my daily life more effectively (P19)

This planned post-retreat integration process was also tied to an **increased motivation for personal growth (4.4.3).** Participants reported that the uncovering of new areas for healing during the integration process spurred them to delve deeper into their self-discovery journeys. This process was further supported by the accountability brought about through sharing their experiences and integration plans with friends and family.

[...] I began to uncover more areas for healing [...] This motivated me to delve deeper and continue on this path. (P12)

Telling my friends and family about my retreat experiences and [my integration plan] [...] made me accountable. Their support encouraged me to continue healing and I even ended up joining more groups and integration circles. (P1)

Lastly, by preparing a post-experience integration plan, participants were able to **strengthen support from their personal networks (4.4.4)**. Informing friends and family about their retreat experiences and subsequent integration plan ahead of time led to increased understanding and support upon returning home. The guidance received during the integration sessions was particularly beneficial for participants in communicating their experiences to those who were less familiar with their journey.

When I came home, my friends… already knowing about my experience… were really supportive and didn’t make me feel weird about my new sort of rituals [...] (P16)

The [integration] sessions with the retreat facilitators were quite useful [...] They helped me communicate my experiences to friends at home, some of whom didn’t really understand my journey. (P13)

#### Part B: perceived ‘most effective’ preparation strategies and barriers

**Figure 2:**
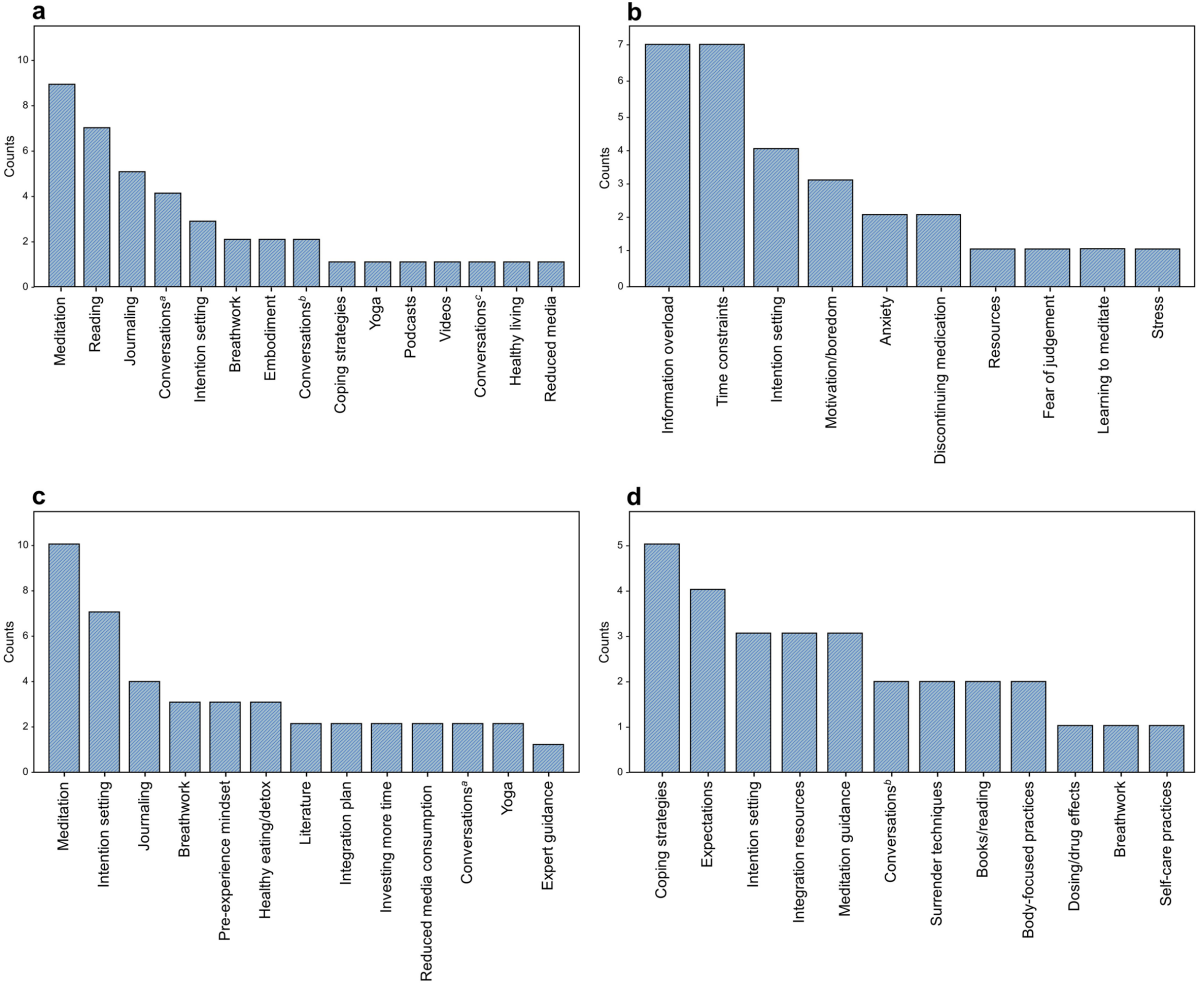
Frequency of participant responses in four domains. (**A**) Effective psychedelic preparation strategies, (**B**) reported hurdles in psychedelic preparation, (**C**) anticipated strategies for future psychedelic experiences, and (**D**) areas requested for additional guidance. For ‘Conversations’: ^a^with experts, ^b^with experienced others, and ^c^ with family/friends.

In the second part of the interview, we identified the strategies that participants deemed most effective for their preparatory regimen preceding their psychedelic experience (i.e. the means they used to achieve an appropriate ‘preparatory set’) and changes they would make in their ‘psychedelic preparation’ for future experiences. In relation to the most effective preparation strategies, ‘Meditation’—specified by participants as techniques including focused-attention, open-monitoring, loving-kindness or self-transcendence, and distinct from breathing or other mind–body practices—was identified by nine participants as the most effective preparatory approach. ‘Reading’ academic literature on psychedelics was another prominent strategy, noted by seven participants, followed by ‘Journaling’ reflectively prior to the psychedelic experience (n = 5). Some participants also favoured ‘Conversations with experts’ (n = 4) and ‘Intention-setting’ (n = 3). While ‘Breathwork’ (n = 2) can often be associated with certain forms of meditation, within this study it was distinctly mentioned as a separate activity that emphasises specialised breathing techniques. Additional preparatory strategies included ‘Embodiment Practices’, which we define here as body-centred exercises distinct from general meditation, and ‘Reduced media consumption’, detailed further in Fig. 2a.

We also elicited details on the variety of obstacles participants encountered during preparation for psychedelic experiences. The most frequently reported obstacles were ‘Information Overload’ and ‘Time Constraints,’ each cited by seven participants (n = 7). Additional challenges included difficulties with ‘Intention-setting’ (n = 4) and ‘Lack of motivation or boredom’ (n = 3). An array of additional barriers emerged, ranging from ‘Anxiety about the impending psychedelic experience’ to ‘Stress associated with reflective practices’, detailed further in Fig. 2b**.**

When asked about prospective modifications to their preparatory regimen planned for future psychedelic sessions, participants articulated a diverse set of preferences. Ten participants intended to prioritise ‘Meditation’ or mindfulness practices. ‘Enhanced intention-setting’ was highlighted by six participants (n = 6), while a focus on ‘Journaling’ was noted by four (n = 4). Additional strategies for future preparation encompassed ‘Breathwork,’ ‘Adjusting one’s pre-retreat mindset,’ and ‘Adopting a healthy diet/detoxification.’ Less frequent but noteworthy were strategies such as ‘Engagement with relevant literature’ and ‘Extended time investment’, detailed further in Fig. 2c.

Participants pinpointed specific domains where additional guidance and information would have enhanced their preparatory process for psychedelic experiences. ‘Formulation of coping strategies and navigation plans’ was cited as a primary concern by five participants (n = 5). Four participants (n = 4) indicated that they would have liked to obtain ‘Clarifications concerning experiential range and expectations.’ Other areas perceived to warrant additional information/guidance included ‘Intention-setting,’ ‘Integration resources,’ ‘Meditation techniques,’ ‘Learning surrender and release techniques,’ ‘Sourcing relevant literature,’ and ‘Understanding dosage parameters and drug effects’, detailed further in Fig. 2d**.**

#### Part C: participant recommendations for structured psychedelic preparation programs

**Figure 3:**
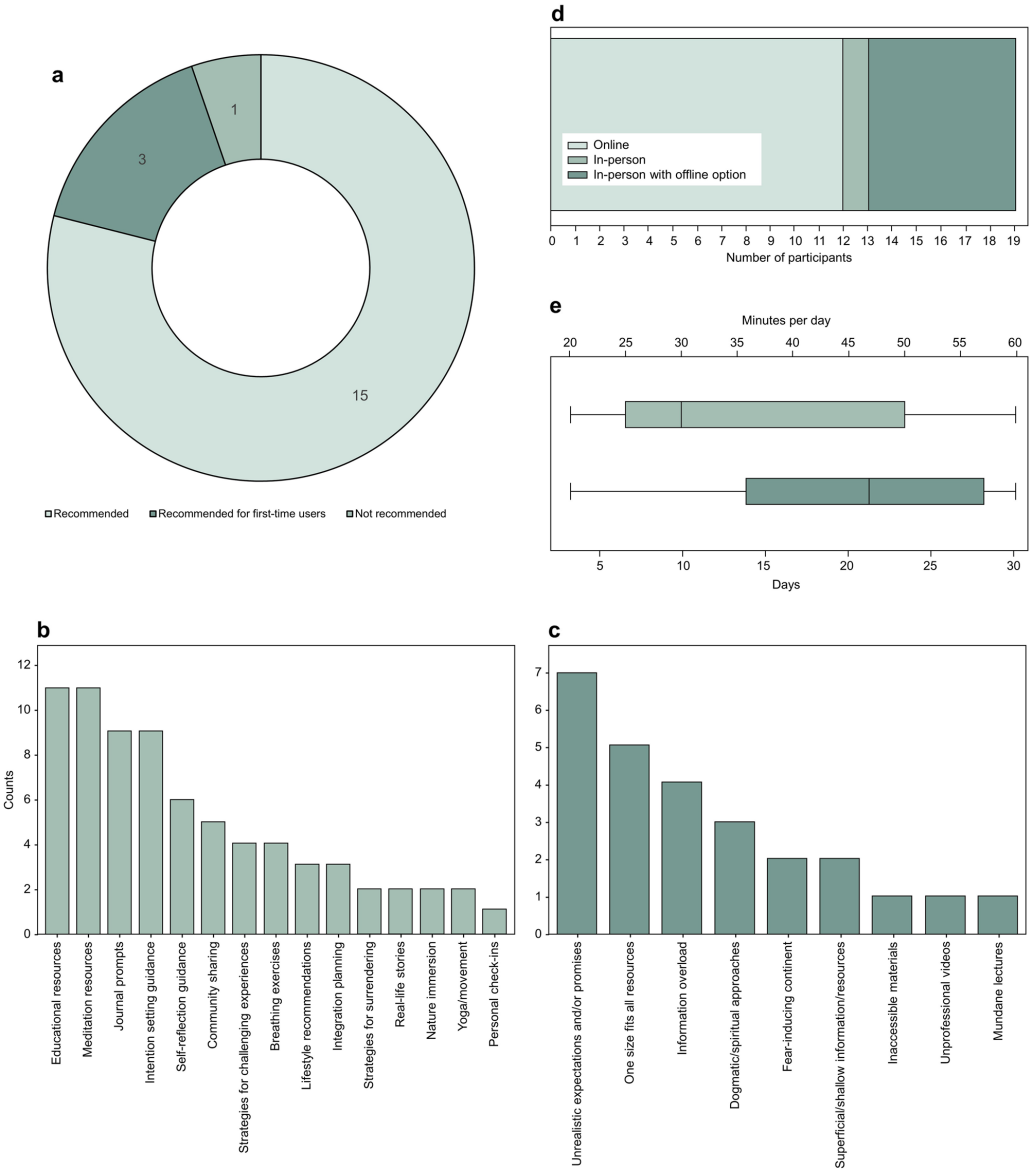
Participant preferences. (**A**) Pie chart showing perceived applicability of a psychedelic preparation intervention (the numbers in the rings refer to the number of participants endorsing each of the three responses). (**B**) Bar chart displaying components recommended for inclusion in the intervention (note: participants could endorse > 1 component). (**C**) Bar chart displaying components recommended for exclusion from the intervention. (**D**) Stacked horizontal bar chart displaying delivery mode preferences among participants. (**E**) Box plots displaying structural preferences, in terms of course duration in days (bottom axis) and time commitment in minutes per day (top axis).

The participants’ perspectives regarding the potential usefulness of a structured preparation program for future participants were elicited in the final part of the interview and are summarised in Fig. 3a**.** The findings indicate that most participants (n = 15; 79%) recommended a preparatory program.

Participant recommendations for key features of the preparation program are summarised in Fig. 3b**.** The most commonly endorsed key features included ‘Educational resources (reading, videos, podcasts)’ (n = 11), ‘Meditation’ (n = 11), and ‘Intention setting guidance’ (n = 9). ‘Journal prompts’ (n = 9) were also frequently mentioned by participants. Other recommended features, with varying percentages and counts, encompassed ‘Community sharing’, ‘Strategies for handling challenging experiences’, ‘Strategies for surrendering’, ‘Lifestyle recommendations (e.g., diet, detox, sleep)’, ‘Post-experience integration planning’, ‘Breathing exercises’, ‘Real-life stories’, ‘Personal check-ins’, ‘Self-reflection’, ‘Nature immersion’, and ‘Yoga/movement’.

Participant recommendations for aspects to avoid in the preparation program are summarised in Fig. 3c. The most commonly advised feature to omit from the program was the ‘Promotion of unrealistic expectations or promises’, mentioned by 7 participants. ‘One-size-fits-all mentality’ or inflexibility was identified as a potential area to avoid by 5 participants, while 4 mentioned ‘Information overload’ as something to be avoided. Other aspects to avoid included ‘Promotion of dogmatic/spiritual approaches’, ‘Fear-inducing content’, ‘Shallow/superficial information/resources’, ‘Inaccessible materials’, ‘Videos with unprofessional or awkward presentation’, and ‘Lectures lacking engagement or failing to hold interest’.

The results revealed that 12 participants expressed a preference for the online format (Fig. 3d). One participant indicated a preference for an in-person format, while six participants preferred an in-person format but mentioned that an offline option would also be acceptable. Among the additional comments provided by participants, two participants expressed a preference for the online format but excluding breathwork activities. Furthermore, five participants suggested the inclusion of live sessions and interactive elements in the course.

Participants’ perspectives on the optimal duration of the course and ideal daily time commitment were collected (Fig. 3e). The desired course duration ranged from 3 to 30 days, with a mean suggestion of 20 days. Daily time commitments varied from 20 to 60 min, with a mean recommendation of 37 min per day.

##### Study 1 summary

The qualitative interviews carried out in Study 1 offered a detailed description of the activities individuals undertake in preparation for psychedelic experiences (Part A), as well as the perceived effectiveness of these methods (Part B), providing an understanding of the practicalities and limitations they encountered. Finally, the interviews explored the participants’ viewpoints on the potential benefits of a structured approach to preparation (Part C), offering insights into how such a framework might be received and its applicability in enhancing the psychedelic experience.

In line with our published theoretical framework on psychedelic preparation, we identified four main themes in participants’ description of preparatory activities in Part A. The Knowledge-Expectation theme describes how participants engaged in thorough personal research into psychedelics, elucidating both immediate and long-term effects. Participants proactively sought resources from scientific literature, books, videos, and podcasts, leading to an improved understanding of psychedelics, aiding in informed decision-making, and reframing perceptions on the safety and historical context of these substances. Additionally, personal accounts, scholarly writings, and online platforms provided insight into the potential subjective effects of psychedelic experiences, facilitating realistic expectations, and underscoring the importance of preparation for their psychedelic encounters. Experiential exercises, including meditation, guided imagery, and sensory deprivation, were also employed to acclimatise participants with psychedelic phenomena. These practices increased confidence, allowed participants to develop coping mechanisms, deepened self-awareness, and provided clarity on altered states of consciousness, helping participants to be better equipped for their psychedelic journeys.

The Psychophysical-Readiness theme emphasised the importance of both mental and physical preparation. Participants emphasised the adoption of various contemplative practices (e.g., focused-attention meditation, meditative-stretch yoga) to promote relaxation and regulate their psychophysical state. These practices reportedly enhanced stress management, fostered a quieting of the mind, and strengthened personal resilience. Meanwhile, cultivating present-moment awareness through practices such as mindfulness meditation, reflective writing, and mindful walking contributed to improved attentional regulation and offered a more neutral observation of thought processes. Furthermore, enhancing body sensations awareness through techniques such as body scan meditations highlighted the importance of mind–body connection, fine-tuning the perception of internal sensations and enhancing control over physical responses. Finally, in optimising physical health, participants gravitated towards balanced diets, regular exercise, and adequate sleep to boost fitness and mood, setting a strong foundation for their psychedelic journey and cultivating resilience to physical discomforts.

The Intention–Preparation theme characterises the introspective process preceding participants’ decision to partake in psychedelics, emphasising the importance of recognising the underlying motivations and setting clear intentions for the experience. Reflecting on their motivations, through methods such as journaling and introspection, participants acquired a deeper self-understanding, and a clearer alignment with their therapeutic objectives. Establishing explicit intentions for their psychedelic journeys, often via journaling or discussion with peers, not only provided a sense of direction during the psychedelic experience, but also heightened the meaningfulness and emotional resonance of the experiences. This foresight further facilitated a structured post-experience integration, as participants could reference their predetermined intentions to better assimilate insights into their daily lives.

The Safety-Planning theme underscores the need for careful preparation and a robust support system during and after the psychedelic experience. Foremost, participants prioritised cultivating trustful relationships with those present during their psilocybin sessions, through dialogues and participatory communal activities. This trust engendered a protective communal network, fostering a profound sense of unity. Vetting the credibility of retreats and staff was important for participants, with many conducting extensive online investigations to ensure safety and reliability, assuaging pre-retreat anxieties. Moreover, to ensure a safe psychedelic experience, participants educated themselves on the structure of the session, the roles of retreat personnel, and developed personal coping techniques, thereby reportedly enhancing their capability to navigate challenging episodes during the experience. Post-experience, a comprehensive integration plan was noted as pivotal. Many participants orchestrated post-retreat integration sessions and established consistent contemplative routines, facilitating reduced post-retreat anxiety, sustained therapeutic benefits, and optimising personal growth trajectories. Communicating their post-retreat integration plans with their personal networks consequently reinforced the supportive framework essential for participants’ post-experience transition and ongoing development.

In the evaluation phase (Phase B), participants underscored several methods they deemed most effective in preparing for their psychedelic experiences. Meditation emerged as the most favoured approach, and, in contemplation of future psychedelic sessions, many indicated an inclination towards adopting regular meditation practices. Reading about psychedelics and journaling were also frequently endorsed. However, participants did face obstacles, notably information overload, time constraints, and difficulties related to setting intentions. Participants clearly expressed a need for additional guidance, particularly on crafting coping strategies and navigating potential challenges. They also sought clarity on what to anticipate and the spectrum of possible experiences.

Phase C assessed participant viewpoints on the potential benefits of a structured preparation program. A considerable majority advocated for the program, though some believed its benefits might be more tailored to first-time users. Participants highlighted several key elements they deemed essential for a preparation course: comprehensive educational resources, meditation practices, and intention-setting guidance took precedence. However, they also cautioned against certain pitfalls, with unrealistic expectations and overgeneralisation being primary concerns. When considering the mode of delivery for such a program, an online format was largely preferred, with some allowing for offline options. Recommendations on the program’s length varied, but there was an average inclination towards a three-week course, with daily commitments ranging around 36 min.

## Study 2

### Methods

#### Recruitment

Participants were recruited on the final day of retreats at one of the collaborating retreat centres. During one of the final sessions, study details were presented to potential participants by author RM. Consenting participants attended a co-design workshop at the end of the retreat. Cumulatively, these sessions included 28 participants and took place in three separate workshops facilitated by RM.

#### Procedures

Three co-design workshops were hosted in-person at the retreat centre, each involving distinct participants from independent retreats. Methodological details are available in Supplementary Material 2A. The overarching goal of this process was to collaboratively generate and refine the selection of activities or components to be included in the initial iteration of the intervention protocol. The collaborative refinement process drew heavily on the findings from the qualitative interviews in Study 1 and our prior framework on psychedelic preparation^25^ (Fig. 3b). Participants were encouraged to use these findings as a guide in identifying and prioritising components for the initial intervention protocol, and were also asked to identify any missing elements. The workshops were designed to balance structured discussion, interactive exercises, prioritisation activities, and individual reflection to encourage the generation of ideas and consensus building. Each session lasted approximately 90 min.

In Workshop 1 the goal was to prioritise key components of a psychedelic preparation intervention. Participants used a dot voting technique to highlight priority components for the proposed intervention. Each component was assigned a priority score (P1, the priority score from Workshop 1), calculated by dividing the total number of priority votes it received by the total number of participants. A P1 score of 1.00 indicates full consensus on the component’s priority.

In Workshop 2 we refined this list using a similar voting procedure to assign priority scores (P2, the priority score from Workshop 2) to the component. Post-voting, participants who marked a component as a priority assessed its feasibility for inclusion in the digital intervention. The feasibility score from Workshop 2 (F2) for each component was determined by dividing the number of feasibility votes it received by the total number of participants. An F2 score of 1.00 signified unanimous agreement on the component’s criticality and feasibility among Workshop 2 participants. Components were ranked based on their F2 scores to streamline the identification of components considered both crucial and feasible for integration by Workshop 2 participants.

The final workshop (Workshop 3) had dual aims: to assess each component for Benefits, Barriers, Risks, and Outcomes, and to develop solutions for identified challenges.

#### Data analysis

The primary data sources from all three workshops was participant feedback, collected as (a) written field notes taken by RM, and (b) the products of written tasks performed by participants throughout the workshops^85,86^. Field notes and participant written material were reviewed and subjected to a rapid thematic analysis by RM and LM^87,88^. This commenced with initial coding, whereby distinct labels representing core ideas were assigned to sections of the notes. These codes were then grouped into broader themes. Qualitative responses were converted systematically into numeric codes and a process of ST-TA^84^ was employed, as in Study 1.

Quantitative data is presented descriptively as counts (or %) and means.

### Results

#### Demographic

Table 2 shows the demographics and psychedelic use variables for the 28 participants who completed this study.

**Table 2:** Sample demographic and psychedelic use variables from Workshops 1–3.

Demographic	Workshop 1 (n = 10)		Workshop 2 (n = 9)		Workshop 3 (n = 9)	
	M	SD	M	SD	M	SD
Age (years)	46.11	8.33	51.13	10.47	51.88	14.32

	n	%	n	%	n	%
Gender						
Male	3	30.00	2	22.22	5	55.56
Female	7	70.00	7	77.78	4	44.44
Education						
High school/college	1	10.00	–	–	–	
Undergraduate	6	60.00	4	44.44	6	66.67
Postgraduate	3	30.00	5	55.56	3	33.33
Ethnicity						
White	8	80.00	6	66.67	6	66.67
Black	1	10.00	–	–	1	11.11
Latino/Hispanic	1	10.00	1	11.11	–	–
Mixed	–	–	1	11.11	2	22.22
Other	–	–	1	11.11	-	-
Religion						
Not religious	7	70.00	6	66.67	6	66.67
Christian	3	30.00	2	22.22	3	33.33
Buddhist	–	–	1	11.11	–	–
Lifetime psychedelic use						
Never before	6	60.00	5	55.56	3	33.34
On 1 occasion only	2	20.00	2	22.22	2	22.22
On 2–5 occasions only	2	20.00	2	22.22	2	22.22
On more than 5 occasions	–	–	–	–	2	22.22

##### Workshop 1: development of a prioritised list of components.

In the individual idea-generation phase, participants generated 31 distinct components, discerned post-hoc through the examination of each participant’s submissions. A significant portion of these could be aligned with the primary components from Study 1, albeit labelled differently. For instance, “Connecting with retreat leaders” from Study 2 aligned with “Personal check-ins” from Study 1. Refer to Supplementary Material 2B for more details.

The group discussions generated a list of 21 agreed components (Table 3), which were presented on a ‘voting form’ which was circulated amongst the participants on which they indicated their priority components . These dot voting results highlighted the top prioritised components (P1) as follows: ‘Reading assignments’ (0.9), ‘Meditation’ (0.8), ‘Journaling’ (0.7), ‘Abstinence from alcohol/drugs’ (0.7), ‘Carving out time post-retreat’ (0.7), ‘Dietary guidance’ (0.7), and ‘Grounding techniques’ (0.6). A detailed list of the prioritised activities can be found in Table 3.

**Table 3:** Prioritisation and ranking of intervention components conducted in Workshop 1 and Workshop 2.

#	Component (theme)	Workshop 1 (n = 10)	Workshop 2 (n = 9)	
		P1	P2	F2
1	Reading assignments (KE)	0.90	0.89	0.89
2	Grounding techniques (PR)	0.60	0.89	0.89
3	Abstinence from alcohol/drugs (PR)	0.70	0.89	0.89
4	Journaling (IP)	0.70	0.67	0.67
5	Carving out time after the retreat (SP)^a^	0.70	0.56	0.56
6	Creating an integration plan (SP)^a^	0.50		
7	Meditation (PR)	0.80	0.67	0.44
8	Resource list (SP)	0.30	0.44	0.44
9	Dietary guidance (PR)	0.70	0.22	0.22
10	Yoga (PR)	0.50	0.22	0.22
11	Connecting with retreat leaders (SP)	0.30	0.67	0.22
12	Panel discussions (KE)^b^	0.20	0.22	0.22
13	Lecture series (KE)^b^	0.10		
14	Physical exercise routines (PR)	0.50	0.11	0.11
15	Nature walks (PR)	0.60	0.22	0.11
16	Quizzes (KE)	0.10	0.11	0.11
17	Holotropic breathwork (KE)	0.50	0.33	0.00
18	Hands-on workshop (KE)	0.30	0.11	0.00
19	Connecting with retreat guests (SP)^c^	0.40	0.22	0.00
20	Group sharing (IP)^c^	0.30		
21	Group discussions (KE)^c^	0.30		

KE: Knowledge-Expectation, PR: Psychophysical-Readiness, IP: Intention-Preparation, SP: Safety-Planning.

^a^‘Carving out time after the retreat’ and ‘Creating an integration plan’ were combined into ‘Planning integration’.

^b^‘Panel discussions’ and ‘Lecture series’ were combined into ‘Expert-led educational sessions’.

^c^‘Connecting with retreat guests’, ‘Group sharing’, and ‘Group discussions’ were combined into ‘Community building & shared experiences’.

Components are organised by theme, with merged components clarified prior to Workshop 2 and indicated with footnotes. Each component aligns with one of the four themes of ‘psychedelic preparedness’ derived from our previous paper^25^: Knowledge-Expectation (KE), Intention-Preparation (IP), Psychophysical-Readiness (PR), and Safety-Planning (SP). In Workshop 1, a ‘priority score’ (P1) was calculated by dividing the total number of priority votes for each component by the number of voters; a score of 1.0 signifies unanimous priority consensus. In Workshop 2, a similar ‘priority score’ (P2) was determined, along with a ‘feasibility score’ (F2), which was derived by dividing the feasibility votes by the total number of participants; a score of 1.0 denotes unanimous criticality and feasibility consensus.

##### *Workshop 2: refinement prioritised* components*.*

Prioritised activities derived from Workshop 1 that were considered to be redundant were merged for clarity prior to Workshop 2: ‘Group discussions’, ‘Group sharing’, and ‘Connecting with retreat guests’ into ‘Community building & shared experiences’; ‘Carving out time’ and ‘Creating an integration plan’ into ‘Planning integration’; and ‘Panel discussions’ and ‘Lecture series’ into ‘Expert-led educational sessions’, which were visually displayed for the dot voting exercise.

Of the 17 components evaluated using the two-step dot voting procedure (taking into consideration both priority and feasibility), the top seven activities receiving the highest F2 scores were ‘Reading assignments’ (0.89), ‘Grounding techniques’ (0.89), ‘Abstinence from alcohol/drugs’ (0.89), ‘Journaling’ (0.67), ‘Planning integration’ (0.56), ‘Meditation’ (0.44), and ‘Resource list’ (0.44). A detailed breakdown encompassing the P2 and F2 scores for each component is provided in Table 3.

##### Workshop 3: evaluation of benefits, barriers, risks, and ideal outcomes and formulation of solutions

Each subgroup critically analysed a subset of the highest ranking F2 components derived from Workshop 2, evaluating their ‘Benefits’, ‘Barriers’, ‘Risks’, and ‘Ideal Outcomes’. The collective insights from these evaluations culminated in a comprehensive group discussion, wherein solutions and strategies were devised to address solutions to the barriers and risks. Table 4 offers a condensed overview of these results.

**Table 4:** A detailed breakdown of the top components from Workshop 3, highlighting their Benefits, Barriers, Risks, Ideal Outcomes, and the Solutions formulated to address challenges.

Component (theme)	Workshop 3				
	Small group discussions				Group Brainstorm
	Benefits	Barriers	Risks	Ideal Outcomes	Solutions
Reading assignments (KE)	Increased understanding of psychedelic experiences Encourages independent research Provides foundational knowledge for discussions	Potential information overload Difficult for non-regular readers Quality and credibility of sources	Misinterpretation of text Over-reliance on a single source Becoming overly critical	Foundational understanding Prepared for informed discussions	Curate concise and vetted reading lists with diverse sources Provide FAQs for each reading to prevent misinterpretations
Grounding techniques (PR)	Offers stabilisation during and after intense experiences Provides tools for emotional and mental regulation	Lack of familiarity with techniques Scepticism about efficacy Difficulty in accessing resources	Ineffectiveness for some individuals Ignoring other necessary interventions	Efficient self-regulation Enhanced mindfulness Equipping participants with tools for during the psychedelic experience and post-retreat life	Introducing various techniques Provide resources or references for continued practice
Abstinence from alcohol/drugs (PR)	Pure experience without influences Resets body chemistry Clarity and focus Eliminates counteractive effects	Potential withdrawal symptoms Social pressures Breaking habits	Abrupt cessation complication Mood disturbances Non-compliance Overcompensation post-retreat	Substance-free entry No drug interactions Clear-headed participation Healthier lifestyle approach	Educate on the benefits of temporary abstinence Provide a support community for those struggling
Journaling (IP)	Personal reflection Intention setting, experience and emotion tracking Encourages self-awareness Post-retreat review tool	Maintaining consistency is difficult Time commitment Over-analysing experiences	Overthinking negatives Misinterpreting writings Overly self-critical behaviour	Personal journey record Regular introspection Tool for post-retreat reflection Enhance personal accountability	Provide structured templates or prompts Regularly remind participants about the purpose and benefits
Planning integration (SP)	Provides dedicated time for processing experiences Ensures intentional reflection and assimilation of retreat learnings Reduces the risk of post-retreat overwhelm Enhances long-term benefits and application of retreat insights	Managing daily commitments with integration time Potential lack of discipline in following through with plans Distractions from daily life and routines Potential for feeling isolated in the process	Inadequate time leading to suppressed or unresolved emotions Misguided or misunderstood integration techniques Potential for neglecting daily responsibilities Over-isolation or excessive introspection	Well-structured post-retreat period maximising benefits Seamless transition from retreat to daily life, retaining retreat insights Enhanced personal growth and understanding from the retreat Continuous integration of retreat learning into daily life	Provide structured post-retreat guidance Organise follow-up group sessions for continued support Offer resources for effective daily-life integration
Meditation (PR)	Emotional regulation Mindfulness Reduces pre-retreat anxiety	Lack of experience Achieving focus difficulty Time commitment Environmental distractions	Unresolved traumas surfacing Misunderstanding purpose Becoming overly introspective	Calm mindset Familiarity with thoughts Handle disturbances	Offer beginner-friendly guided sessions Start with short sessions and progressively increase Offer support for surfacing traumas and misunderstood objectives
Resource list (SP)	Provides attendees with credible, trustworthy preparation material Makes research easier Standardised information disseminated to attendees	Overwhelming volume of resources Inconsistency in quality or relevance of resources Potentially biased or one-sided material	Misinterpretation or misinformation Over-reliance on a singular source Information overload	Informed and well-prepared attendees Cultivation of a culture of continuous learning Encouragement of diverse perspectives	Periodically update and vet resources Provide summaries or overviews for each resource Encourage participants to seek multiple perspectives

KE: Knowledge-Expectation, PR: Psychophysical-Readiness, IP: Intention-Preparation, SP: Safety-Planning.

Each component aligns with one of the four themes of ‘psychedelic preparedness’ derived from our previous paper^25^: Knowledge-Expectation (KE), Intention-Preparation (IP), Psychophysical-Readiness (PR), and Safety-Planning (SP).

#### Study 2 summary

Building on the findings from Study 1, three workshops in Study 2 focused on identifying additional potential intervention components and refining this list to a set of essential activities for a preparation-phase psychedelic intervention.The aim was to identify activities that resonated most with the participants, ensuring that the intervention started on a strong footing with components of high perceived value.

The first workshop resulted in 31 distinct components which were prioritised through dot voting. ‘High priority’ components included ‘Reading assignments’, ‘Meditation’, and ‘Journaling’.

In Workshop 2 we identified the highest priority and most feasible intervention components, such as ‘Grounding techniques’, ‘Abstinence from alcohol/drugs’, and ‘Planning integration’ emerged as highly prioritised and feasible.

In Workshop 3 we finalised the components for a preparation intervention through additional detailed evaluation of the intervention activities identified in the Workshops 1 and 2. The central importance of activities like ‘Meditation’ and developing/accessing a ‘Resource list’ were confirmed and strategies for implementing these activities were identified.

Throughout the workshops, the activities were categorised into the four main themes from Study 1: Knowledge-Expectation (KE), Intention-Preparation (IP), Psychophysical-Readiness (PR), and Safety-Planning (SP), which were themselves conceptualised in this way based on our previous psychometric work on preparation for psychedelic experiences. This thematic approach helped in organising the components systematically and provided a coherent framework for the intervention. The workshops successfully identified and refined the highest-ranking activities, ensuring that the final intervention was built on components that were not only valued by participants but also practical and effective in the context of psychedelic preparation.

### General discussion

This paper describes the co-production of the elements of a Digital Intervention for Psychedelic Preparation (DIPP). Preparation is recognised as a critical component of psychedelic-assisted interventions, although we are not aware of any structured intervention describing a set of theoretically coherent components that are likely to be valued by participants and feasible to implement. Our study aimed to fill this gap by drawing directly on the experience of participants who were undergoing psychedelic ‘treatment’ in the context of a retreat setting, locating these findings within a recently described theoretical framework developed through extensive consultation with experts and psychometric evaluation^25^.

Guided by this framework^25^, Part A of Study 1 identified specific preparatory behaviours and strategies prior to psychedelic engagement which could be understood in terms of the superordinate themes of Knowledge–Expectation, Psychophysical–Readiness, Intention–Preparation, and Safety–Planning. Subsequent evaluation in Part B underscored meditation as a predominant preparatory activity. However, participants indicated facing barriers, notably in the form of information overload. A prominent theme was the need for structured guidance, particularly concerning the development of coping strategies and expectation management. In Part C, the cohort overwhelmingly supported a structured preparatory regimen, underscoring the imperative of robust educational materials, consistent meditation practices, and clear intention-setting guidelines. Digital delivery was the predominant preference, with an optimal duration proposed to span approximately three weeks.

Through sequential co-design workshops in Study 2, we refined a list of components tailored for DIPP. Workshop 1 identified primary components, while Workshop 2 prioritised them based on feasibility. Key components integrated were ‘Reading assignments’ (KE), ‘Grounding techniques’ (PR), ‘Abstinence from alcohol/drugs’ (PR), ‘Journaling’ (IP), ‘Planning integration’ (SP), ‘Meditation’ (PR), and a the provision of a ‘Resource list’ (SP). In Workshop 3, subgroups extensively evaluated these components, considering their benefits, barriers, risks, and ideal outcomes.

The results from both studies were used to design a provisional 21-day self-led, digital intervention that aims to increase psychedelic preparedness. We have structured our description around the first 6 items of the 12-item TIDieR checklist^68^, a tool designed for enhancing the clarity of intervention reporting. The detailed interpretation of each item for this intervention is displayed in Supplementary Table 3B. The latter 6 items will be expounded upon post the pilot testing of the intervention.

#### Intervention description

Developed using a person-centric, systematic methodology and informed by the MRC complex intervention framework, DIPP is anchored in the four-factor model of psychedelic preparedness^25^. Iterative modifications to its content and design were informed by qualitative data from Study 1 interviews and feedback gathered during Study 2’s co-design workshops.

**Figure 4:**
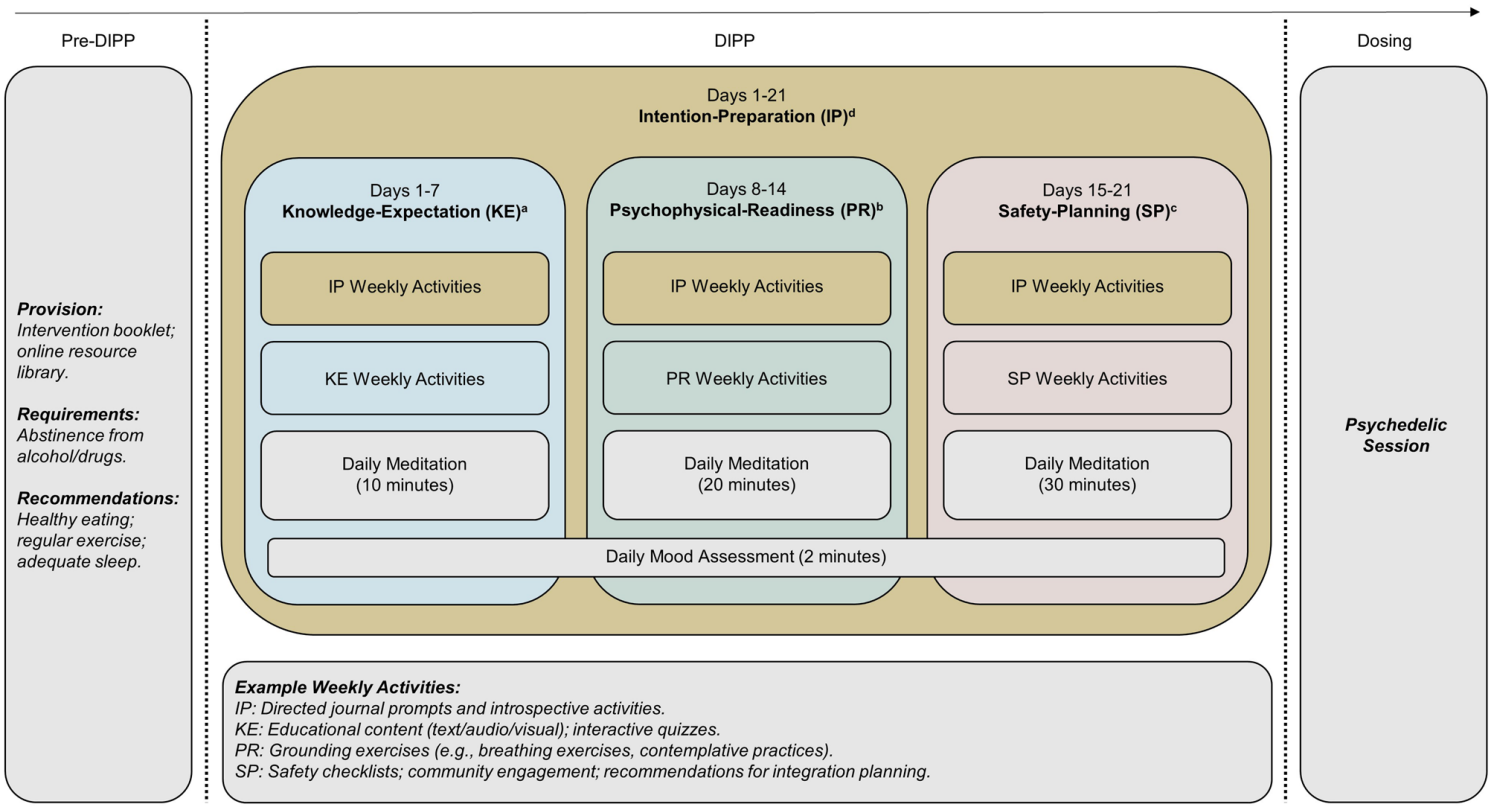
Structure and thematic modules of DIPP. The intervention comprises four primary thematic modules: Knowledge-Expectation (KE) from day 1–7, Psychophysical-Readiness (PR) from day 8–14, and Safety-Planning (SP) from day 15–21, with a concurrent Intention-Preparation (IP) module spanning across the entire period. Each week includes a selection of module specific activities. Each day includes guided meditation sessions and a brief daily mood assessment. ^a^Aims to deepen participants’ knowledge of psychedelics and their potential effects and outcomes. ^b^Aims to equip participants with tools and techniques to optimise mental and physical readiness for the psychedelic experience. ^c^Aims to create a secure environment for psilocybin administration and to facilitate effective post-session integration. ^d^Aims to guide participants in defining their motivations and setting clear intentions for psychedelic use.

**Figure 5:**
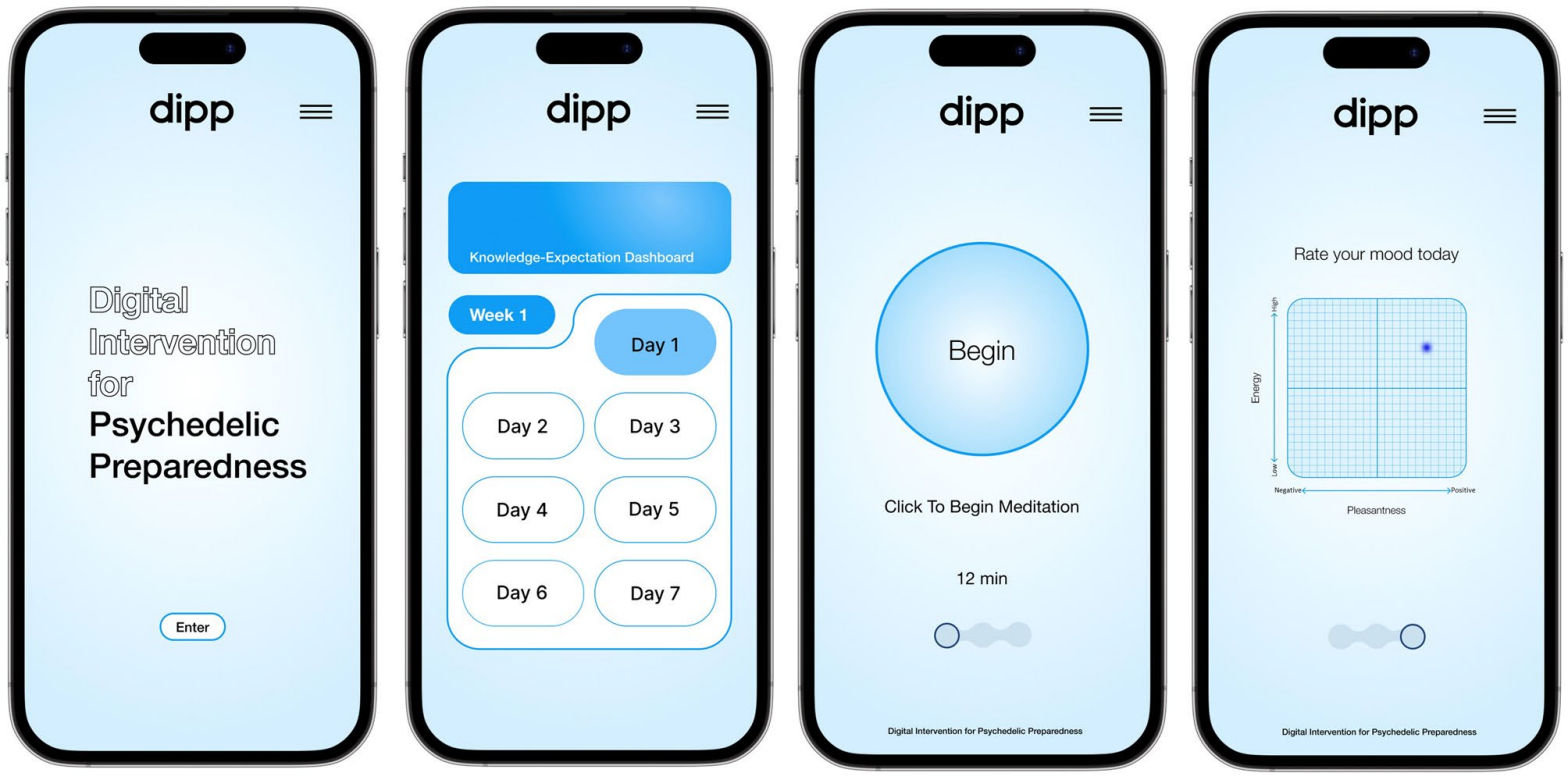
Screenshots of DIPP.

DIPP spans three weeks, with three of the four modules completed sequentially and the fourth (Intention-preparation) completed in parallel throughout the three weeks (as illustrated in Fig. 4). Figure 5 shows several screenshots from the DIPP platform, designed and developed by asgc studio. The discrete components of DIPP resonate with the current trend towards modular designs in the development of complex interventions^89–94^. Drawing upon insights from Studies 1 and 2, each module introduces specialised preparatory activities (detailed in Table 4), which participants engage with at their own pace during the designated week. More information on module structure can be found in Supplementary Material 3C.

In both studies, ‘Meditation’ was consistently identified as a valuable preparatory component for psychedelic experiences, which corresponds with previous research^95–98^. For example, a recent longitudinal study found that more days of loving-kindness or compassion meditation practice prior to a psychedelic experience seemed to buffer against certain challenging psychedelic experiences^99^. Other research also suggests that tools or techniques to calm the mind are helpful during challenging psychedelic experiences^18,20^, which points to the potential benefit of using different meditation practices as a preparatory component for psychedelic experiences. Accordingly, our intervention includes a fifth module comprising structured daily meditation sessions (described in forthcoming paper). Although mechanisms are unknown, it is possible that the development of acceptance, presence, and compassion, alongside the ability to decenter and confront challenges, may be particularly beneficial for psychedelic preparedness^97^. However, the precise role of meditation as a preparatory tool for enhancing psychedelic therapy remains under-explored and warrants further investigation.

The modules outlined above would themselves be preceded by a prep-DIPP introductory program booklet detailing the course structure and objectives. This booklet will include instructions for the daily meditation sessions that are integral to the intervention. In addition, an online ‘Resource library’ (SP) will be made available, offering supplementary materials corresponding to each of the four thematic modules. Participants are mandated to ‘Abstain from drugs and alcohol’ (PR) for the duration of the 21-day intervention. Concurrently, they are advised to adopt a balanced diet, engage in regular exercise, and maintain sufficient sleep. To monitor participants’ mental states, a brief online mood assessment is administered daily.

The DIPP framework is designed to be versatile, applicable across different user groups such as those in psychedelic-assisted psychotherapy, recreational users, and participants at retreats. It is also intended to be compatible with a range of psychedelics. Nevertheless, it is crucial to recognise that the framework’s content requires customisation to cater to the unique needs of each user group and the specific characteristics of the psychedelic in question. While the overall delivery method remains constant, the Knowledge-Expectation component must be precisely tailored. This entails that the psychoeducational materials provided must be specifically aligned with the effects and properties of the particular psychedelic substance being used. The content within other modules of the framework, as well as the meditation materials, is structured to be consistent across different psychedelic substances. This uniformity ensures a stable foundation for the framework’s application, irrespective of the psychedelic used.

#### Strengths and limitations

In accordance with the MRC guidelines, our study systematically involved stakeholders throughout the intervention development process. This engagement enhanced the depth and breadth of our contextual understanding, ensuring the intervention’s relevance and alignment with genuine user needs^46,100,101^. This active involvement of stakeholders emphasises the significance of participatory research in intervention development^102^, bridging theoretical constructs with practical utility. By adopting this method, we advocate for interventions that are both methodologically sound and relevant to real-world contexts. The MRC highlights the need for intervention development to be dynamic, iterative, and receptive to change while focusing on future evaluations and implementations^52^. Iterative cycles, characterised by stakeholder feedback, subsequent refinements, and evaluations, are integral to this approach. As we advance to the piloting phases of our study, continuous stakeholder engagement will remain pivotal. Our collaborative approach clearly privileged the experiential accounts and knowledge of ‘experts-by-experience’. However, we acknowledge that an important test of the value of these intervention components will rely on direct evidence of improved health or mental well-being outcomes in those who undergo a structured intervention containing these components relative to credible control interventions.

It is important to acknowledge other limitations inherent in our sampling strategy. Our stakeholders predominantly identified as White (Study 1: 73.7%; Study 2: averaging 67.42% across workshops). This underrepresentation of other ethnicities is a limitation relevant to the majority of psychedelic-assisted psychotherapy studies^103,104^. Furthermore, while there is a balanced distribution in terms of gender and a broad spectrum of ages, our participants were generally highly educated. In terms of psychedelic use, many had limited or no prior experiences. Hence, while the feedback from our stakeholder group was valuable, it does not include the broader range of experiences of those who could benefit from the intervention. Future research efforts should prioritise diversifying stakeholder involvement to ensure a more comprehensive understanding and generalisability of interventions. Additionally, the participant age range in our studies—minimum 37 years in Study 1, with a median of approximately 50 in Study 2—is a significant limitation regarding representativeness. This skew towards older demographics may not reflect the preparatory needs and digital engagement preferences of younger psychedelic users^105,106^. Given their potential for distinct technological engagement and content interaction patterns, the applicability of DIPP to this group remains uncertain^107^. Future research should prioritise inclusivity of younger demographics to ensure DIPP’s adaptability and relevance across a wider age spectrum, thereby enhancing its external validity.

While online interviews (Study 1) offer wider accessibility and flexibility than face-to-face interviews, and also potentially allow for more candid discussions on sensitive topics due to the potential reduced discomfort between the interviewer and interviewee^108^, challenges such as biases introduced by technology access, establishing trust, and assessing participant safety are documented concerns^109,110^. A primary consideration in this method of data collection is the potential influence of recollection bias on the accuracy of participants’ responses^111–114^. Nonetheless, the interviews were conducted on average of 3.16 months (SD = 2.09) post-retreat, attenuating concerns of pronounced memory deterioration. Further, the consistent and precise nature of participants’ accounts regarding their preparatory actions underscores their capacity to recall specific details with clarity.

Beyond recollection bias, our sample’s exclusive focus on participants reporting a ‘significant change’ post-retreat introduces potential confirmation bias. This bias manifests in two distinct ways. Firstly, those with transformative, positive experiences may retrospectively ascribe greater efficacy to their preparation strategies, inflating their perceived significance. On the other hand, the experiences of participants who encountered challenging or negatively perceived significant changes are not represented in our sample. Such individuals might retrospectively attribute lesser efficacy to the same preparation strategies. This omission suggests that our findings might skew towards a positive interpretation of the effectiveness of some preparatory modalities, overlooking the nuanced experiences of those who had less favourable outcomes. Therefore, caution is warranted in generalising these findings as universally indicative of the effectiveness of the identified preparatory methods. Future research should aim to include a more diverse range of experiences, encompassing both positive and challenging outcomes, to provide a more balanced and comprehensive understanding of the intervention’s impact.

The participatory nature of the workshops (Study 2) promoted a synthesis of diverse perspectives, lending depth and a diversity of user-perspectives to the intervention design. It also overcomes the recall bias in Study 1. Nevertheless, the group dynamics inherent in workshops are known to influence individual contributions^115,116^, with dominant voices potentially overshadowing more reticent participants^117,118^. Additionally, while the interactive format stimulates rich discussions, it may also lead to convergent thinking^119–121^, potentially limiting the breadth of ideas presented. To address these potential limitations, we employed various strategies. Firstly, we emphasised the significance of active participation to all participants at the start of each workshop. Notably, participants shared a pre-established familiarity due to their attendance at the psilocybin retreats, fostering a harmonious group dynamic without apparent dominance. To encourage diverse ideation and mitigate the risk of convergent thinking, we integrated individual tasks within the workshop sessions, alongside smaller group and whole-group discussions. These measures aimed to stimulate autonomous thinking and inclusive discourse, contributing to the development of a comprehensive intervention.

The focus on participants from psilocybin retreats also introduces potential limitations regarding the intervention’s broader applicability in clinical and research contexts. Notably, retreat attendees may constitute a distinct group, characterised by specific characteristics (e.g. high income) that is not representative of those who might undergo psychedelic treatment in clinical settings. Their motivations were not assessed, but these are likely to differ from clinical populations who might seek psychedelic treatment for a health condition. Moreover, the clinical population may encompass individuals with specific psychiatric profiles, diverse therapeutic expectations, and the need for tailored therapeutic interventions—elements potentially underrepresented in the retreat cohort. The structured ambiance of a retreat, often enriched with spiritual or communal undertones, might also dictate distinct preparatory requirements compared to more neutral clinical or research environments. It is imperative to recognise, however, that the study’s foundation was informed by prior research conducted on a broader spectrum of psychedelic users^25^. This previous research culminated in the development of the four-factor model, encapsulating elements deemed generally pertinent across diverse settings. The comprehensive nature of this dataset underpins our assertion that the intervention retains the potential for adaptability beyond retreat contexts. Hence, while the present study offers useful insights from the retreat milieu, our overarching framework of psychedelic preparedness, rooted in a more heterogeneous sample, suggests that the intervention, with judicious adaptations, remains promising for a spectrum of clinical and research applications.

While our approach emphasises the accessibility and convenience of digital platforms, there is a legitimate concern that such methods might inadvertently underemphasise the relational aspects critical to psychedelic experiences, particularly those pertaining to the bond with guides and fellow participants. The trustful relationships fostered in traditional, in-person settings are central to the effectiveness of psychedelic-assisted psychotherapy^26^, as highlighted in our ‘Subtheme 4.1: Cultivating Trustful Relationships’. The potential risk that digital preparation could undermine these essential relational dynamics needs to be acknowledged. As such, while we advocate for the integration of digital methods in preparatory stages, it is crucial to maintain a balance that ensures the preservation and emphasis of these interpersonal connections.

In concluding the initial development phase of DIPP, it is vital to acknowledge and report the relevant uncertainties that may influence its future effectiveness and applicability. Notably, the intensity of the intervention, optimal modes of delivery, specificity of materials, and procedural nuances remain to be finely calibrated. Additionally, the type of setting most conducive to the intervention—whether in clinical environments, retreats, or independent use—requires further elucidation. These uncertainties present avenues for subsequent research. Moreover, they serve as a critical consideration for practitioners integrating this intervention within their healthcare contexts, who must be cognisant of these variables when tailoring the intervention to individual user needs. Recognising these uncertainties not only transparently positions the current state of the intervention but also strategically informs the next phases of research and application, ensuring the DIPP’s evolution is informed by both empirical evidence and practical experiences.

#### Conclusion

This work details a novel 21-day digital intervention developed to enhance psychedelic preparedness (DIPP). Grounded in the four-factor model of psychedelic preparedness^25^ and informed through a person-centric approach, DIPP integrates distinct thematic modules that address the multidimensional aspects of preparation. The intervention incorporates daily meditation sessions, a technique consistently highlighted in our findings and the broader literature for its potential in optimising psychedelic experiences. While the feedback and design have been comprehensive, the study recognises the limitations posed by the sample demographics and the specific context of psilocybin retreats.

### Supplementary Information

Supplementary Information.

## Data Availability

The data generated and/or analysed during this research will be made available upon request by contacting the corresponding author.
