# Supplementary material for: Development of a digital intervention for psychedelic preparation (DIPP)
Source: Sci Rep. 2024 Feb 19;14:4072. doi: 10.1038/s41598-024-54642-4 (PMC10876638; doi:10.1038/s41598-024-54642-4)
Supplement: Supplementary file 1 — Supplementary Information. [file 41598_2024_54642_MOESM1_ESM.pdf]

Development of a Digital Intervention for Psychedelic Preparation (DIPP)

Rosalind G. McAlpine[1]*, Matthew D. Sacchet[2], Otto Simonsson[3], Maisha Khan[1], Katarina Krajnovic[1], Larisa Morometescu[1], Sunjeev K. Kamboj[1]

[1] Clinical Psychopharmacology Unit, Clinical, Educational and Health Psychology, University College London
[2] Meditation Research Program, Department of Psychiatry, Massachusetts General Hospital, Harvard Medical School
[3] Department of Neurobiology, Care Sciences and Society, Karolinska Institutet

* Corresponding author: rosalind.mcalpine.18@ucl.ac.uk

Supplementary Material

1A. CONTEXTUAL INFORMATION - PSILOCYBIN RETREAT CENTRES

This section provides detailed information about the psilocybin retreat centres involved in our study. While these centres vary in location and specific approach, they share commonalities in their operational framework, target population, and overall objectives. The centres are unified by their focus on offering ‘healing’ experiences, primarily to individuals seeking therapeutic benefits from psilocybin for conditions such as depression and anxiety. Notably, these centres predominantly cater to first-time psilocybin users. While there are differences in the specific programs of each retreat, the centres were selected for collaboration based on the similarities in their program structures and types of support offered. It is acknowledged that these variations could potentially influence participants' experiences and outcomes.

Participant Population and Screening: Each centre conducts its own screening process, aiming to create a safe and supportive environment. The primary criterion for admission is the absence of current diagnoses or family history of psychosis or schizophrenia. This screening ensures that the participants are suitable for the psilocybin experience, primarily focusing on individuals seeking healing from various psychological conditions.

Program Structure: Despite slight variations, the retreats generally follow a similar structure. This structure encompasses a preparation day at the beginning, followed by the dosing days, and concludes with an integration day. The dosing days are interspersed with various workshops, including meditation, yoga, and breathwork. These activities are designed to enhance the psychedelic experience and promote introspection and personal growth.

Sharing Circles and Participant Interaction: A critical component of these retreats is the incorporation of sharing circles. These circles provide participants with a platform to express their feelings and discuss their psilocybin experiences. This practice fosters a sense of community and mutual support, enhancing the overall therapeutic experience.

Facilitation and Support: The retreats do not include specific psychotherapy sessions; however, they are facilitated by trained individuals who offer non-directive support. These facilitators play a crucial role during the group ceremonies, which often include live music. They are also available to assist participants with practical needs and provide grounding support when necessary.

2A. CO-DESIGN WORKSHOP METHODOLOGICAL DETAILS

Workshop 1

The objective of Workshop 1 was to develop a prioritised list of components.

Participants were first introduced to the findings from Study 1 (**Figure 3b**). Based on these findings, they were instructed to delineate potential components for the intervention. They were encouraged to draw from the findings of Study 1 and, importantly, to supplement these with any additional components they deemed relevant to the intervention's objectives. Each participant separately recorded their inputs, either in writing or through diagrams.

Next, a group discussion was held to facilitate knowledge sharing and collaboration. During this session, participants evaluated and discussed the proposed components, resulting in a compiled list, presented on a single sheet of paper.

The dot voting method was then employed [70–72], whereby each participant was provided with ten coloured stickers to mark the components they prioritised as most important to include in the intervention.

Workshop 2

The objective of Workshop 2 was to further refine the set of components identified in Workshop 1. This refinement was driven by the goal of achieving consistent exposure to intervention components across the intervention while mitigating the risk of diminishing engagement due to excessive repetition and general participant burden [73–75]. Informed by these considerations and underscored by the significance of user-centric design [76], we decided to select the top seven activities from this workshop to take forward into Workshop 3.

The workshop began with a presentation of the findings from Workshop 1, followed by a group discussion focusing on each of the components. This was to ensure all participants had a clear understanding of each component's objectives and principles. Emphasis was placed on assessing the relevance of each component for the digital intervention and the practicality of incorporating it.

Subsequent to the discussion, a refined evaluation procedure was implemented via a two-step dot voting procedure. The necessity for this modification became apparent in Workshop 1, where the initial assessment only gauged the critical importance of components without considering participants' opinions on the feasibility of embedding these components into a digital intervention. To address this, a secondary assessment step was incorporated. On a single piece of paper, participants marked activities they viewed as ‘priority’ with a green sticker. Components marked as a priority were then further evaluated by participants for their suitability within a digital intervention framework, with a secondary green sticker indicating practical ‘feasibility’.

Workshop 3

The two primary objectives of Workshop 3 were: (1) To evaluate each component in terms of its Benefits, Barriers, Risks, and projected Outcomes (BBRO) and (2) To formulate solutions for the challenges identified.

The session started with a summary of the findings from Workshop 1 and Workshop 2. After a group discussion, participants were segmented into smaller teams. Each team was allocated a set of components from the top eight identified in previous workshops. Under the BBRO framework, teams listed specific points for their respective components, categorising them accordingly.

Following the subgroup evaluations, participants reconvened to collaboratively discuss and propose potential solutions and strategies to address the identified challenges.

2B. W1 IDEA GENERATION PHASE

Activities derived from Study 2 (Workshop 1) thematic analysis, discerned post-hoc through the examination of each participant's submissions. Corresponding Study 1 components are displayed to the right.

#	Study 2: components (theme)	Study 1: corresponding components
1	Reading assignments (KE)	*Educational resources*
2	Journalling (IP)	*Journal prompts*
3	Meditation (PR)	*Meditation materials*
4	Physical exercise routines (PR)	*Yoga/movement*
5	Holotropic breathwork (KE)	*Breathing exercises*
6	Grounding techniques (PR)	*Strategies for handling challenging experiences*
7	Carving out time after the retreat (SP)[a]	*Post-experience integration planning*
8	Yoga (PR)	*Yoga/movement*
9	Dietary guidance (PR)	*Lifestyle recommendations*
10	Abstinence from alcohol/drugs (PR)	*Lifestyle recommendations*
11	Creating an integration plan (SP)[a]	*Post-experience integration planning*
12	Resource list (SP)	*Educational resources*
13	Connecting with retreat leaders (SP)	*Personal check-ins*
14	Hands-on workshop (KE)	•
15	Group discussions (KE)[c]	*Community sharing*
16	Lecture series (KE)[b]	*Educational resources*
17	Nature walks (PR)	*Nature immersion*
18	Quizzes (KE)	*Educational resources*
19	Connecting with retreat guests (SP)[c]	*Community sharing; Real-life stories*
20	Group sharing (IP)[c]	*Community sharing; Real-life stories*
21	Sound baths (PR)	•
22	Dance and movement (IP)	*Yoga/movement*
23	Documentary viewings (KE)	*Educational resources*
24	Panel discussions (KE)[b]	*Educational resources*
25	Virtual reality experiences (KE)	•
26	Sleep hygiene (PR)	*Lifestyle recommendations*
27	Vision board creation (IP)	•
28	Affirmation crafting (IP)	•
29	Guided visualisations (IP)	•
30	Arts and crafts (IP)	•
31	Buddy system (SP)	*Personal check-ins;*

3A. GUIDED

GUIDED - A guideline for reporting for intervention development studies:

Item description	Explanation	Page in manuscript
1. Report the context for which the intervention was developed.	Understanding the context in which an intervention was developed informs readers about the suitability and transferability of the intervention to the context in which they are considering evaluating, adapting or using the intervention. Context here can include place, organisational, and wider sociopolitical factors that may influence the development and/or delivery of the intervention.	5
2. Report the purpose of the intervention development process.	Clearly describing the purpose of the intervention specifies what it sets out to achieve. The purpose may be informed by research priorities, for example those identified in systematic reviews, evidence gaps set out in practice guidance such as The National Institute for Health and Care Excellence or specific prioritisation exercises such as those undertaken with patients and practitioners through the James Lind Alliance.	3
3. Report the target population for the intervention development process.	The target population is the population that will potentially benefit from the intervention – this may include patients, clinicians, and/or members of the public. If the target population is clearly described then readers will be able to understand the relevance of the intervention to their own research or practice. Health inequalities, gender and ethnicity are features of the target population that may be relevant to intervention development processes.	27
4. Report how any published intervention development approach contributed to the development process	Many formal intervention development approaches exist and are used to guide the intervention development process (e.g. 6Squid or The Person Based Approach to Intervention Development). Where a formal intervention development approach is used, it is helpful to describe the process that was followed, including any deviations. More general approaches to intervention development also exist and have been categorised as follows: Target Population-centred intervention development; evidence and theory-based intervention development; partnership intervention development; implementation-based intervention development; efficacy based intervention development; step or phase-based intervention development; and intervention-specific intervention development. These approaches do not always have specific guidance that describe their use. Nevertheless, it is helpful to give a rich description of how any published approach was operationalised.	3
5. Report how evidence from different sources informed the intervention development process.	Intervention development is often based on published evidence and/or primary data that has been collected to inform the intervention development process. It is useful to describe and reference all forms of evidence and data that have informed the development of the intervention because evidence bases can change rapidly, and to explain the manner in which the evidence and/or data was used. Understanding what evidence was and was not available at the time of intervention development can help readers to assess transferability to their current situation.	24
6. Report how/if published theory informed the intervention development process.	Reporting whether and how theory informed the intervention development process aids the reader's understanding of the theoretical rationale that underpins the intervention. Though not mentioned in the e-Delphi or consensus meeting, it became increasingly apparent through the development of our guidance that this theory item could relate to either existing published theory or programme theory.	24
7. Report any use of components from an existing intervention in the current intervention development process.	Some interventions are developed with components that have been adopted from existing interventions. Clearly identifying components that have been adopted or adapted and acknowledging their original source helps the reader to understand and distinguish between the novel and adopted components of the new intervention.	n/a
8. Report any guiding principles, people or factors that were prioritised	Reporting any guiding principles that governed the development of the application helps the reader to understand the authors' reasoning behind the decisions that were made. These could include the examples of particular populations whose views are being considered when designing the intervention, the modality that is viewed as being most appropriate, design features considered important for the target population, or the	24-25

when making decisions during the intervention development process.	potential for the intervention to be scaled up.	
9. Report how stakeholders contributed to the intervention development process.	Potential stakeholders can include patient and community representatives, local and national policy makers, health care providers and those paying for or commissioning health care. Each of these groups may influence the intervention development process in different ways. Specifying how differing groups of stakeholders contributed to the intervention development process helps the reader to understand how stakeholders were involved and the degree of influence they had on the overall process. Further detail on how to integrate stakeholder contributions within intervention reporting are available.	5, 15-16
10. Report how the intervention changed in content and format from the start of the intervention development process.	Intervention development is frequently an iterative process. The conclusion of the initial phase of intervention development does not necessarily mean that all uncertainties have been addressed. It is helpful to list remaining uncertainties such as the intervention intensity, mode of delivery, materials, procedures, or type of location that the intervention is most suitable for. This can guide other researchers to potential future areas of research and practitioners about uncertainties relevant to their healthcare context.	18-23
11. Report any changes to interventions required or likely to be required for subgroups.	Specifying any changes that the intervention development team perceive are required for the intervention to be delivered or tailored to specific sub groups enables readers to understand the applicability of the intervention to their target population or context. These changes could include changes to personnel delivering the intervention, to the content of the intervention, or to the mode of delivery of the intervention.	26
12. Report important uncertainties at the end of the intervention development process	Intervention development is frequently an iterative process. The conclusion of the initial phase of intervention development does not necessarily mean that all uncertainties have been addressed. It is helpful to list remaining uncertainties such as the intervention intensity, mode of delivery, materials, procedures, or type of location that the intervention is most suitable for. This can guide other researchers to potential future areas of research and practitioners about uncertainties relevant to their healthcare context.	27-28
13. Follow TIDieR guidance when describing the developed intervention.	Interventions have been poorly reported for a number of years. In response to this, internationally recognized guidance has been published to support the high-quality reporting of public health interventions. This guidance should therefore be followed when describing a developed intervention.	24
14. Report the intervention development process in an open access format.	Unless reports of intervention development are available, people considering using an intervention cannot understand the process that was undertaken and make a judgement about its appropriateness to their context. It also limits cumulative learning about intervention development methodology and observed consequences at later evaluation, translation and implementation stages. Reporting intervention development in an open access (Gold or Green) publishing format increases the accessibility and visibility of intervention development research and makes it more likely to be read and used. Potential platforms for open access publication of intervention development include open access journal publications, freely accessible funder reports or a study web-page that details the intervention development process.	

*e.g. if the item is reported elsewhere, then the location of this information can be stated here.

3B. TIDieR

Template for Intervention Description and Replication (TIDieR) checklist:

Item No	TIDieR Item	Current Intervention Description
Brief name		
1	Provide the name or a phrase that describes the intervention	Self-led, digital psychedelic preparedness intervention.
Why		
2	Describe any rationale, theory, or goal of the elements essential to the intervention	Rationale: The states induced by classic psychedelics necessitate a comprehensive psychological preparation to ensure participant safety and optimise therapeutic outcomes. Despite the acknowledged importance of this preparation, current approaches often lack consistent standardisation.<br><br>Theory: The core premise of our intervention is based on the hypothesis that an evidence-backed, individualised preparation can enhance the therapeutic efficacy of psychedelics while minimising risks. By improving participants' understanding, ensuring readiness, guiding introspective focus, and emphasising safety, the intervention aims to provide an optimal setting for effective psychedelic experiences. Integrating self-directed strategies with clinician-led methods is believed to better accommodate individual variability in preparation needs.<br><br>Goal: The primary objective is to establish a methodical, evidence-based, and individualised preparation process for psychedelic interventions. Key components from our four-factor model include:<br>❖ Knowledge-Expectation: Educating participants about psychedelics to align expectations with likely outcomes.<br>❖ Psychophysical-Readiness: Preparing participants mentally and physically to effectively manage the nuances of the psychedelic experience.<br>❖ Intention-Preparation: Guiding introspective reflection to ensure participants approach the experience with clear objectives.<br>❖ Safety-Planning: Implementing measures to ensure a secure and supportive environment throughout the psychedelic session.<br>To achieve these goals, the intervention employs various tools and strategies, such as structured educational materials, meditation practices, and intention-setting protocols, each developed based on empirical evidence and participant feedback.
What		
3	Materials: Describe any physical or informational materials used in the intervention, including those provided to participants or used in intervention delivery or in training of intervention providers. Provide information on where the materials can be accessed (such as online appendix, URL)	Format & Structure: The intervention is delivered through an online platform, allowing individual user access via unique login credentials. This digital format ensures consistent delivery and facilitates tracking of individual progress.<br><br>Thematic Modules: The intervention is organised into a series of three one-week thematic modules:<br>❖ Week 1: 'Knowledge-Expectation'<br>❖ Week 2: 'Psychophysical-Readiness'<br>❖ Week 3: 'Safety-Planning'<br>An overarching module, 'Intention-Preparation,' is interwoven throughout the entire three-week period, reinforcing its pervasive importance in the psychedelic preparation process.<br><br>Daily Protocols: Each day comprises:<br>❖ A session of guided meditation, backed by instructional materials (detailed in a forthcoming paper).<br>❖ A list of auxiliary activities resonating with the weekly theme. Participants can engage with these activities flexibly, as per their convenience within the respective week.<br>❖ A concise online mood assessment, facilitating daily monitoring of participants' psychological states.

		Introductory Materials: Before initiating the intervention, participants are furnished with an introductory program booklet. This document elucidates the course's architecture and objectives and provides comprehensive instructions for the daily meditation exercises integral to the intervention (details to be expanded upon in a forthcoming paper).<br><br>Resource Library: An online resource repository is available to participants, stocked with supplementary materials tailored to each of the four thematic modules. This library serves as a reference hub, enabling participants to delve deeper into topics of interest.<br><br>Lifestyle Guidelines: During the 21-day intervention span, participants are explicitly instructed to refrain from consuming drugs and alcohol. Furthermore, they receive guidelines promoting a balanced diet, regular physical activity, and adequate sleep to optimise the intervention's efficacy.<br><br>Accessibility: All the intervention materials, including the introductory booklet, daily protocols, and online resource library, are hosted on our dedicated platform. Access details and further specifics will be provided in the appendices of the forthcoming study.
4	Procedures: Describe each of the procedures, activities, and/or processes used in the intervention, including any enabling or support activities	Intervention Initiation:<br>❖ Participants register on the online platform and receive unique login credentials.<br>❖ Upon initial login, they are directed to download and review the introductory program booklet to familiarise themselves with the course structure and objectives.<br>❖ They are also guided on how to navigate the online resource library and access supplementary materials as needed.<br><br>Daily Protocol:<br>❖ Each day begins with participants logging in to the platform.<br>❖ They are prompted to engage in the guided meditation session and are provided access to the instructional materials.<br>❖ Post-meditation, they receive a list of auxiliary activities specific to the week's theme, allowing them to choose based on interest and convenience.<br>❖ By the day's end, participants complete the online mood assessment, providing immediate feedback on their psychological state.<br><br>Weekly Thematic Engagement:<br>❖ Each week is categorised by its thematic module. The platform's dashboard or main page highlights the theme prominently, ensuring participants are aligned with the week's focus.<br>❖ The online resource library is curated to emphasise materials corresponding to the current week's theme, facilitating deeper engagement.<br><br>Lifestyle Monitoring and Adherence:<br>❖ Throughout the 21-day period, participants receive periodic reminders, both within the platform and via email notifications, emphasising the importance of abstaining from drugs and alcohol.<br>❖ They also get periodic tips and guidelines on maintaining a balanced diet, engaging in physical activity, and ensuring adequate sleep.<br><br>Feedback and Support:<br>❖ An integrated support feature on the platform allows participants to raise queries or seek clarifications on the materials or procedures.<br>❖ Weekly feedback forms enable the intervention team to gather insights and address any concerns promptly.

Who provided		
5	For each category of intervention provider (such as psychologist, nursing assistant), describe their expertise, background, and any specific training given.	1. Academics and Clinical Psychologists:<br>Expertise:<br>❖ Specialised in the study and clinical application of psychedelics and their therapeutic implications.<br>❖ Proficient in developing structured educational content based on empirical evidence and best practices in psychedelic therapy.<br>Background:<br>❖ Typically hold a Ph.D. in Clinical Psychology, Neuroscience, or a related field.<br>❖ Possess extensive experience in psychedelic research, both in clinical and academic settings.<br>Specific Training:<br>❖ All contributing academics and clinical psychologists have been a part of interdisciplinary conferences and workshops focused on psychedelic therapy and its advancements.<br>❖ They have undergone peer-review processes and collaborations to ensure the content is comprehensive, accurate, and relevant for the intervention's intended audience.<br><br>2. Meditation Practitioners:<br>Expertise:<br>❖ Experienced in guiding meditation sessions tailored for introspection, mental preparedness, and grounding.<br>❖ Understand the nuances of meditative practices as they relate to psychedelic experiences, ensuring participants are mentally aligned and centred.<br>Background:<br>❖ Certified from recognized meditation or mindfulness institutions or have significant lineage-based training.<br>❖ Boast a track record, often spanning a decade or more, of leading meditation sessions, workshops, and retreats.<br>Specific Training:<br>❖ All meditation practitioners involved in content creation have been briefed on the specifics of the psychedelic intervention and its objectives.<br>❖ They have collaborated with the clinical psychologists to tailor the meditation guides, ensuring they align with the themes of the intervention and meet the unique requirements of preparing individuals for psychedelic experiences.
How		
6	Describe the modes of delivery (such as face to face or by some other mechanism, such as internet or telephone) of the intervention and whether it was provided individually or in a group	Platform Delivery: The primary mode of delivery for the intervention is via an online digital platform. Participants access the intervention content, materials, and activities through this platform.<br><br>Delivery Mechanism:<br>❖ Internet-Based: The intervention's core content and resources are hosted on a dedicated online platform. This allows for uniform content presentation and easy accessibility for all participants from any location, provided they have internet connectivity.<br>❖ Automated Email Notifications: Periodic reminders, tips, and guidelines are disseminated to participants through email notifications to reinforce adherence to the intervention's protocols and lifestyle guidelines.<br><br>Nature of the Intervention:<br>❖ Individually Tailored: While the content remains consistent for all participants, the digital nature of the intervention allows for individualised progress tracking. Each participant has a unique login, and their engagement with the intervention is self-paced and self-led. This ensures that the experience is tailored to each individual's pace and preferences.

		Group Interaction:<br>❖ The intervention is primarily designed for individual engagement, ensuring personal introspection and reflection. However, given the digital format, there is potential (if deemed beneficial in the future) to incorporate group forums or discussion boards for participants to share experiences or seek peer support.

3C. Module Structure Information

The modules are structured as follows:

- Week 1/Module 1 (Days 1-7): Knowledge-Expectation (KE) provides an in-depth education on psychedelics. Through tailored 'Reading assignments', it assesses participants' current understanding of these substances and sets their expectations regarding both immediate and prolonged outcomes of their use.
- Week 2/Module 2 (Days 8-14): Psychophysical-Readiness (PR) focuses on evaluating participants' psychological and physiological preparedness for the multifaceted nature of psychedelic experiences. This module emphasises the participants' capacity to confront potential emotional or physical challenges, fostering an acceptance of unforeseen elements of the experience, and building trust in their inherent ability to navigate these challenges skilfully. The main activities taught here are 'Grounding techniques', to provide participants with tools to use before, during and after the psychedelic experience.
- Week 3/Module 3 (Days 15-21): Safety-Planning (SP) emphasises the imperative of creating a safe and supportive environment during the psychedelic session. Key aspects include cultivating trust with attendants, confirming substance safety, preparing acquaintances for possible behavioural or emotional changes, and formulating contingency strategies for any post-experience challenges. 'Integration planning', within this context, predominantly addresses strategies for post-experience reflection and coping, ensuring participants are well-prepared for the aftermath.
- Weeks 1-3/Module 4 (Days 1-21): Interwoven throughout the duration of the program is the Intention-Preparation (IP) module. Through reflective 'Journaling' exercises, this module delves into the underlying motivations and intentions for psychedelic-use, whether they be oriented towards self-exploration, therapeutic applications, or spiritual growth.
